# Microliths in the South Asian rainforest ~45-4 ka: New insights from Fa-Hien Lena Cave, Sri Lanka

**DOI:** 10.1371/journal.pone.0222606

**Published:** 2019-10-02

**Authors:** Oshan Wedage, Andrea Picin, James Blinkhorn, Katerina Douka, Siran Deraniyagala, Nikos Kourampas, Nimal Perera, Ian Simpson, Nicole Boivin, Michael Petraglia, Patrick Roberts

**Affiliations:** 1 Department of Archaeology, Max Planck Institute for the Science of Human History, Jena, Germany; 2 Department of History and Archaeology, University of Sri Jayewardenepura, Gangodawila, Nugegoda, Sri Lanka; 3 Department of Geography, Royal Holloway, University of London, London, England, United Kingdom; 4 Research Laboratory for Archaeology and the History of Art, University of Oxford, Oxford, England, United Kingdom; 5 Department of Archaeology, Government of Sri Lanka, Colombo, Sri Lanka; 6 Biological and Environmental Science, University of Stirling, Stirling, Scotland, United Kingdom; 7 Centre for Open Learning, University of Edinburgh, Edinburgh, Scotland, United Kingdom; 8 School of Social Science, The University of Queensland, Brisbane, Queensland, Australia; 9 Department of Anthropology and Archaeology, University of Calgary, Calgary, Canada; 10 Department of Anthropology, National Museum of Natural History, Smithsonian Institution, Washington, D.C., United States of America; Monash University, AUSTRALIA

## Abstract

Microliths–small, retouched, often-backed stone tools–are often interpreted to be the product of composite tools, including projectile weapons, and efficient hunting strategies by modern humans. In Europe and Africa these lithic toolkits are linked to hunting of medium- and large-sized game found in grassland or woodland settings, or as adaptations to risky environments during periods of climatic change. Here, we report on a recently excavated lithic assemblage from the Late Pleistocene cave site of Fa-Hien Lena in the tropical evergreen rainforest of Sri Lanka. Our analyses demonstrate that Fa-Hien Lena represents the earliest microlith assemblage in South Asia (*c*. 48,000–45,000 cal. years BP) in firm association with evidence for the procurement of small to medium size arboreal prey and rainforest plants. Moreover, our data highlight that the lithic technology of Fa-Hien Lena changed little over the long span of human occupation (*c*. 48,000–45,000 cal. years BP to *c*. 4,000 cal. years BP) indicating a successful, stable technological adaptation to the tropics. We argue that microlith assemblages were an important part of the environmental plasticity that enabled *Homo sapiens* to colonise and specialise in a diversity of ecological settings during its expansion within and beyond Africa. The proliferation of diverse microlithic technologies across Eurasia *c*. 48–45 ka was part of a flexible human ‘toolkit’ that assisted our species’ spread into all of the world’s environments, and the development of specialised technological and cultural approaches to novel ecological situations.

## Introduction

In the last decade, growing archaeological and palaeoenvironmental evidence has documented the use of tropical rainforest resources by *Homo sapiens* in several locations in South Asia, South East Asia, and Melanesia between 45,000 and 36,000 years ago [1–8]. There have also been more tentative suggestions of tropical rainforest adaptations by our species at earlier dates in Africa [9–11] and Sumatra [12]. Scholars had previously considered these environments to be barriers to human forager occupation, due to the scarcity of carbohydrate-rich plants, limited fat and protein-rich fauna, and difficulties of movement and thermoregulation [13–16]. Instead, most discussions of the evolution and migration of our species focused on the medium- and large-sized game available in grassland or savanna settings [17–19] or protein rich maritime resources available in coastal settings [20,21]. Archaeological evidence for the recurrent exploitation of tropical rainforests has also indicated that these environments played a central role in human adaptations [5–8]. The wide use of ecological settings by our species demonstrates increased levels of ecological plasticity, enabling the spread of human populations into a diversity of ‘extreme’ environments during its expansion within and beyond Africa [22,23].

**Fig 1:**
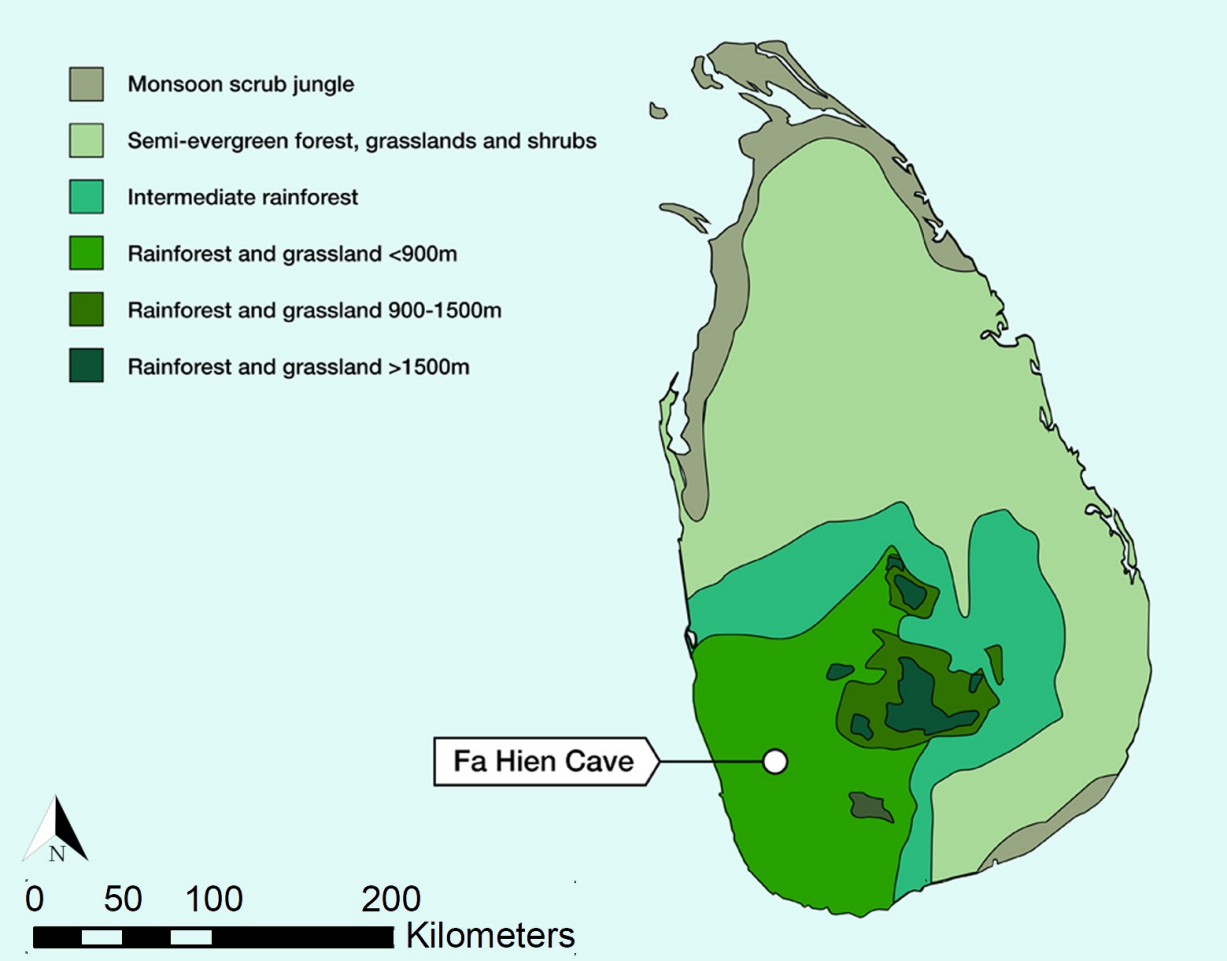
Map of the position of Fa-Hien Lena relative to Sri Lanka’s vegetation zones.

The island of Sri Lanka, at the southern tip of South Asia, has emerged as a particularly important area for the investigation of prehistoric hunter-gatherer adaptations and technological strategies used in tropical rainforest ecosystems. Caves and rockshelters excavated in the modern Wet Zone rainforest of Sri Lanka since the 1950s (Fig 1) have yielded long stratigraphic sequences, with well-preserved organic plant and animal remains in Late Pleistocene and Holocene contexts [1,24,25]. The earliest human fossils of South Asia are found in the Sri Lankan caves and rockshelters, in levels dated to *c*. 45,000–36,000 cal. years BP [26,27]. Stable isotope analysis of human and animal tooth enamel, alongside zooarchaeological and archaeobotanical analysis [25,26], has highlighted that these human foragers relied almost entirely on rainforest resources for their subsistence needs between 36,000–3,000 cal. years BP [5,7]. What is less clear, however, is the range of technological strategies that these populations used to enable their dedicated rainforest subsistence practices, and how adaptations may have varied through time. Analysis of bone tools found at the site of Batadomba-lena has suggested that they were used as components of composite projectiles, in traps, or even as freshwater snail picks [28]. Stone tools, predominately made from small flakes of quartz, occur in much greater abundance in these early rainforest occupation sites but their interpretation has typically focused on simplistic typological comparisons, entraining them into competing models of human dispersal [29–31]. More detailed appraisal of lithic technology from Batadomba-lena suggests that further evaluation of technological variability is warranted within the early rainforest occupation sites of Sri Lanka [32], alongside a wider framework for interpretation of the origins and uses of these lithic industries.

Here, building on the new multidisciplinary analysis of the Fa-Hien Lena sequence [26], we report the first detailed analysis of lithic technology from the site Fa-Hien Lena, based upon excavations conducted in 2009, 2010, and 2012 [26] (Fig 1). The earliest lithic industries at this site are associated with the oldest skeletal evidence for *H*. *sapiens* in South Asia, found in a rainforest context [26]. Below we review historical and current debate regarding microlithic stone tool industries as well as available evidence for the colonisation of rainforest habitats by *H*. *sapiens*. Following a description of the stratigraphy and chronology of Fa-Hien Lena, we present a detailed assessment of the nature of lithic technology and examine patterns of change through time. We place these results in their regional context, through comparisons with the well-studied Sri Lankan site of Batadomba-lena [32,33], as well as more broadly across South Asia. We also evaluate our results within wider discussions of the emergence of microlithic technologies and their role in adaptation to diverse ecological contexts, with a particular focus on rainforest environments.

## Background

### Defining the microlithic

Defining microlithic technology is not straightforward, as no single ‘one-size-fits-all’ definition can readily encompass all archaeological assemblages to which the term has been applied without losing analytical value. Half a century ago Clark defined a series of technological modes [34] as part of a framework rooted in the European record that would help organise and understand global patterns in Palaeolithic technological variability. In this system, microlithic technologies comprised ‘Mode 5’, yet this definition conflated aspects of production, focusing on small flakes and blades as blanks for retouched tools, and use, with the implication that these tools were used in composite, hafted tools. While undoubtedly this definition has proven to be both useful and influential, including its application within South Asia, a growing body of evidence suggests that definitions of the microlithic based on their expression in Europe may not be appropriate for other regions of the world [35–40].

The creation of criteria for identifying microlithic technologies based upon characteristics of individual archaeological sites or regions is problematic. Pargeter [40] breaks the focus on small, backed bladelets into its three component elements focused purely on stone tool reduction (excluding assumptions of use): 1) systematic production of small flakes from fine grained stone; 2) use of backing (abrupt) retouch, including the production of geometric forms; and 3) bladelet production from prismatic cores. More recently, Pargeter and Shea [41] examined long-term trends in the practice of miniaturisation of lithic technology and presented a number of useful criteria from archaeological, experimental and ethnographic studies to clarify and constrain the use of terms such as microlithic (summarised in Table 1).

**Table 1:** Criteria for identifying miniaturised lithic reduction practices (after Pargeter and Shea [41]). Given that the term ‘microlith’ is often interchangeable with backed geometrics in the South Asian Palaeolithic literature, disambiguation is important.

Type	Criteria
Targeted Production of Small Tools	As a reductive medium, flaking at many scales can produce small artefacts. Identification of dedicated focus on small tools can include the following: a) Demonstrate that small size is not a restriction of clast size availability through raw material availability; b) Preclude impact of reduction intensity as key driver of size by illustrating systematic core reduction and management practices amongst small artefacts; c) Exhibit selection of small flake blanks for retouched tools.
Small tool sizes	Inter-regional comparisons suggest artefacts <50mm can reflect systematic production of small tools.
Production Methods	Freehand flaking becomes more difficult amongst cores <20mm, whereas bipolar flaking can remain productive for cores <10mm.
Microflake *vs* Microblade	Both flake and blade production can feature amongst small tool technologies, rather than either being a prerequisite; while bladelets may increase the production of cutting edge on regular sized/shaped blanks, the application of laminar methods may be limited by differences in utility in individual artefacts requiring higher replacement rates and higher risks of bending fractures, leading to a preference for homogeneous materials.
Tool use	Backing is a prominent form of retouching of small tools, and may improve the strength of hafted pieces; however, unretouched small flakes can also make efficient hand tools and hafted tool inserts.

Here, we retain the term microlithic, and use it specifically to refer to stone tool technologies dedicated to the production of small lithic artefacts, conforming to the criteria proposed by Pargeter and Shea [41] (Table 1), rather than on the explicit focus on small backed tools. Indeed, microlithism need not necessarily include backed tools [41–43]. Following the demonstration of the bimodal distribution of blade sizes in southern India [44], we employ a 40mm size threshold and describe flakes, blades (bladelets), and retouched tools smaller than 40mm as microlithic. In a similar manner, cores that have been systematically exploited to produce blanks below the 40mm threshold are described as microlithic. While the appearance of bladelets and backed artefacts can be a feature of microlithic technologies, their absence is not considered critical for attributing lithic assemblages to microlithic technologies.

#### Antiquity of the microlithic in South Asia

In the middle of the 20th century, the African terminology for describing prehistoric stone tool industries was employed in South Asia, differentiating the Early, Middle and Later Stone Age (e.g. [45]). Within this scheme, Later Stone Age assemblages broadly corresponded to microlithic technologies. The adoption of the European terminology of Lower, Middle, Upper Palaeolithic, and Mesolithic in South Asia from the 1970’s onwards led to a more direct parallel being drawn between the microlithic industries of South Asia and the Mesolithic of Europe. This led to the longstanding use of the term ‘Mesolithic’ to encompass microlithic industries (e.g. [1,46]), as well as the argument for a recent antiquity of microlithic technologies, thought typically to date to the early Holocene or the terminal Pleistocene. Given the scarcity of chronometric dates, this viewpoint persisted in spite of evidence for microlithic assemblages older than 25 ka in India [47] and as early as *c*. 30 ka in Sri Lanka [1].

During the past decade a growing number of sites associated with chronometric ages have clearly demonstrated the Pleistocene antiquity of microlithic assemblages in South Asia. In India, these include studies from South India (e.g. Jurreru Valley ~35 ka [48–49]), Central India (e.g. Mehtakheri ~44 ka [50]; Patne >25 ka [47]), West India (e.g. Buddha Pushkar ~28 ka [51]), North India (e.g. Middle Son Valley 55–47 ka [52]) and East India (e.g. Kana ~42 ka [53]). Recent reappraisal of the chronology of microlithic assemblages from Sri Lanka, clearly demonstrates a comparable antiquity for microlithic industries at Batadomba-lena (~36 ka) [25] and Kitulgala Beli-lena (~33 ka) [1,5,54]. Critically, renewed dating programs at these Sri Lankan sites are extending the chronological range of these industries, including at Fa-Hien Lena, the site with the earliest confirmed ages for human occupation on the island [5].

Historically, microlithic technologies in South Asia were argued to have developed locally from a distinct Upper Palaeolithic antecedent (e.g. [55]). However, the recognition of a shared African ancestry for all modern humans, as opposed to a strong multi-regional model, has led to a focus on microlithic industries as potential markers of the rapid expansion of *H*. *sapiens* populations through coastal environments or grassland corridors [29,50,56]. This is, in part, due to the place of microliths within a package of behaviours thought to be unique to ‘modern’ humans, emerging in Africa by 80–60 ka [56–58]. Such models, rooted in mtDNA studies of contemporary populations and simplistic lithic comparison [57,58], have been subject to sustained critique on a number of grounds. Most crucially here, technological diversity of microlithic assemblages between Africa and Asia was ignored in favour of asserting a typological ubiquity [32,33]. An absence of microlithic industries around much of the Indian Ocean Rim also makes suggestions of cultural inheritance between Africa and South Asia difficult to support [30]. Nuclear genome research, as well as fossil discoveries across Asia [59,60], has also complicated the association of microlith toolkits with the first members of our species in different parts of the world. Finally, the appearance of backing and microliths in Uluzzian industries in Italy, which are amongst the earliest microlithic industries outside of Africa and are associated with *H*. *sapiens*, clearly suggests that microliths were not the sole preserve of foragers in woodland and savanna settings [61–63].

In their review of the global appearance of backed microliths, Clarkson and colleagues [39] argue that convergent evolution in different environmental and cultural settings, not a single origin and dispersal [58], offers a better explanation of the global origins of microliths. They argue that microliths offered a range of functional advantages, including transportability, efficiency of raw material use, ease of manufacture and maintainability, and advantages specific to backed pieces, including standardisation, haftability and reliability, that would promote their adoption by numerous, unrelated populations. In several regions in India, the innovation of microlithic tool kits appears to be rooted in previous Middle Palaeolithic technologies [44,52]. In both these Indian examples, the appearance of microliths is linked to the combination of increasing demographic pressures and worsening climatic conditions. Elsewhere, it has been argued that demographic pressures have played a major role in technological development among our species [64]. In contrast to the contexts in which they have been found in the rest of the world, in Sri Lanka microlithic technologies occur in tropical rainforest environments, often argued to be more stable ecosystems in the face of climatic change [1,46]. However, despite the significance of microliths in these contexts, detailed discussions of temporal changes in their production, morphology, and the context of their use have been limited to isolated studies [1,32].

### Fa-Hien Lena Cave

**Fig 2:**
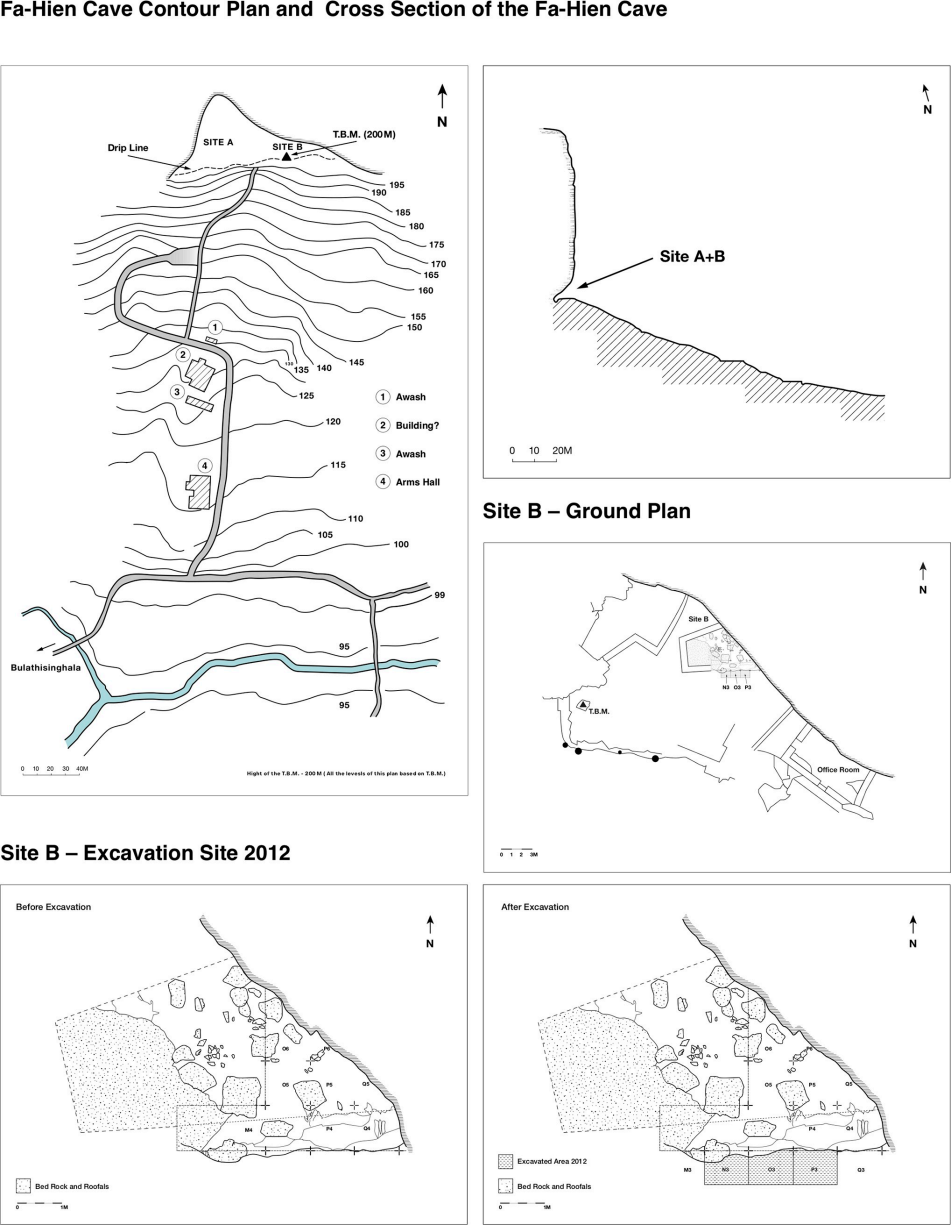
Fa-Hien Lena site plan. A) Elevation map of Fa-Hien Lena and surroundings. Levels based on T.B.M -200m; B) Section plan of Fa-Hien Lena; C) Ground plan of site B; D -E) Ground plan of the excavated area in 2009 and 2012.

Fa-Hien Lena cave, one of the largest caves in Sri Lanka, is situated in Yatagampitiya village (80°12’E, 6°38’N), near the Bulathsinhala Divisional Secretariat Division in the Kalutara District of the Western Province (Fig 1), at approximately 130 meters above mean sea level. It is a shallow rockshelter containing prehistoric habitation, situated next to a large domed cave [24,46,65]–formed in an almost vertical southwest-facing cliff of Proterozic gneiss of the Highlands Complex [66]. The humus-stained cliff containing these caves drops from a forested summit to the banks of a small stream. The caves are situated at the gradient break between the steep rock cliff and the lower gradient colluvium slope (Fig 2). Large boulders on the forested colluvial slopes below the caves provide evidence for relatively recent rockfall and retreat of the steep gneiss cliff [24,46,62,65].

The first excavations at Fa-Hien Lena Cave occurred between 1986–1988 in two main areas, Cave A and Shelter B, with the aim of understanding the cultural sequence of the cave (Fig 2) [24]. Cave A, the larger one of the two, was first excavated to a depth of over 6 m. It yielded deposits of what appears to be roof-fall flakes and decaying bedrock throughout the profile, without any definitive trace of early human habitation [24,26]. Shelter B, a smaller subsidiary rockshelter located approximately 20m east of the main Cave A chamber, proved to be far more productive. Excavations conducted in 1986 identified five strata, with excavation reaching bedrock [1,24,46]. In 2009, 2010, and 2012 fieldwork at Shelter B was carried out aiming at enhancing the stratigraphic and chronological resolution achieved by Wijeyapala in the 1980s, and to excavate the lower portions of the deposit to bedrock [24]. The most recent excavations were undertaken in a 300cm × 100cm x 220cm trench positioned in the east-west direction of the southern profile of 1986 excavation (Fig 2).

### Stratigraphy and chronology

**Fig 3:**
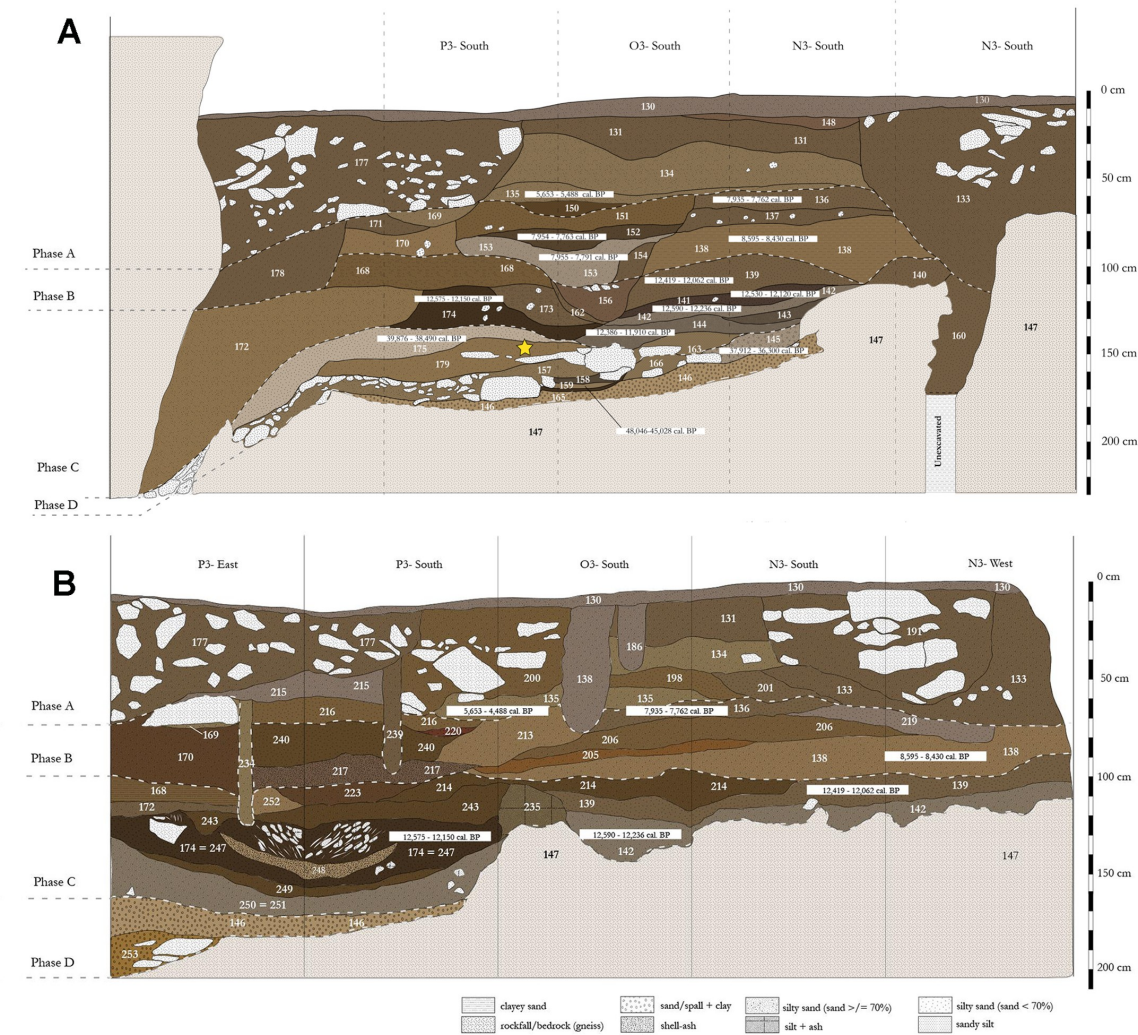
Fa-Hien Lena site stratigraphy. A) South wall end of the 2010 excavation taken from Wedage et al. [26]; B) South wall end of the 2012 excavation. Colours represent Munsell colour values of sediments. Phases D, C, B, and A, and their associated radiocarbon age brackets (see also [26]). Yellow star shows human fossil identified by Kennedy [27], see also Wedage et al. [26].

The stratigraphic and chronological analysis of Fa-Hien Lena has been developed by Wijeyapala [24], Kennedy [27], and Wedage et al. [26] over the course of the last three decades. The sediment fill of Fa-Hien Lena Shelter B consists of *c*. 170cm of stratified detrital sediments deposited on the heavily weathered and phantomed gneiss bedrock (Fig 3). Based on recent re-dating efforts, these deposits date from as early as *c*. 48,000–45,000 cal. years BP [26]. The fossils found at Fa-Hien Lena [1,24,27], and their associated material culture, thus represent the earliest definitive evidence for *H*. *sapiens* in Sri Lanka and South Asia more broadly. Radiocarbon dates cluster into four distinct age ranges, separating the stratigraphy into four distinct phases, each correlating with a major period of human occupation of the cave (Fig 3) (Table 2) [26].

**Table 2:** Calibrated radiocarbon dates for Fa-Hien Lena published in Wedage et al. [26]. All samples have been calibrated using the OxCal 4.3 software and IntCal13 calibration curve [67–69]. *Sample rejected, see [26] for further details.

Phase	Context	Sample	Calibrated (cal. years BP)	Uncalibrated dates (years BP)
A	107	BYP2010/CX NE/N-4, O-4, 107	4,422–4,248	3,870 ± 30
	2	B-N5-2	5,594–5,322	4,750 ± 60
	31/32/135	135	5,653–5,488	4,820 ± 30
	116	BYP 2010 CX NE/0-6, 0–6, 116 middle	5,710–5,482	4,800 ± 40
B	3	B-M6-2	7,916–7,570	6,850 ± 80
	136	136	7,935–7,762	6,970 ± 30
	51/152	152	7,954–7,763	6,990 ± 40
	52/153	153	7,955–7,791	6,900 ± 40
	3a	B-N6-2a	8,020–7,794	7,100 ± 60
	51/152	152	8,180–8,020	7,240 ± 40
	138	138	8,595–8,430	7,720 ± 40
C	109	BYP2010/CX NE/N-4, O-4, 109	12,096–11,768	10,150 ± 40
	119	BYP 2010 CX NE/O-4, P-4, 119	12,380–11,844	10,250 ± 40
	144/161/164	144	12,386–11,910	10,290 ± 40
	139/140	139	12,419–12,062	10,350 ± 40
	141	141	12,530–12,120	10,340 ± 40
	237	237	12,549–12,131	10,390 ± 40
	174/246	174	12,575–12,150	10,440 ± 40
	142	142	12,590–12,236	10,430 ± 40
D	4	B-M7-3	29,126–27,872*	24,470 ± 290
	4	B-N7-3	34,656–33,686	30,060 ± 290
	118	BYP 2010 NE/O-4, 118	36,136–35,191	31,750 ± 190
	4a	B-M7-5	37,912–34,764	32,060 ± 630
	145	145	37,912–36,300	32,890 ± 240
	5	B-M6-6	38,826–35,828	33,070 ± 630
	108	BYP 2010 CX NE/O-4, P-4, 108	38,333–36,690	33,220 ± 240
	175	175	39,876–38,490	34,600 ± 320
	110	BYP2010/CX NE/N-4, O-4, 110	42,036–40,980	36,910 ± 300
	126	BYP 2010 CX NE/ O-4, 126F	42,228–41,258	37,230 ± 310
	159	159	48,046–45,028	43,000 ± 720

Phase D (Late Pleistocene) deposits begin with pebbly clayey loams with angular gneiss spalls, followed by sub-horizontal layers of dark, sandy silt and laminated ash deposits interlayered with at least two distinct accumulations of angular gneiss blocks. Faunal remains (28.7% of which are burned/calcined) and artefacts (shell beads, ochre fragments, bone points, quartz flakes) are abundant throughout. Micromorphological analysis revealed various coarse-grained microfacies (laminated and phosphatised ash intercalated with gneiss pebbles; structureless pebbly ash; microaggregated organic loam; imbricated microbreccia with charred organic matrix), all containing plentiful, well-preserved human occupation debris (charcoal, burned shell and bone, palm and many other phytoliths, quartz microflakes probably derived from lithic knapping) [26]. Phase D sediments record intermittent/episodic human occupation, from *c*. 48,000 to 34,000 cal. years BP, and colluvial inwash during a period of structural instability of the cave walls and ceiling. Human-mediated sediment inputs came from hearths–some *in situ–*and the cooking and consumption and discard of food remains (including the nuts of a prominent tropical and sub-tropical tree species *Canarium* sp., other, as yet unidentified plant remains, snails adapted to rainforest environments, monkeys and other predominantly arboreal mammals [26], and, possibly, the use of palm fronds for the construction of artefacts, e.g. mats, baskets and other, similar containers, as inferred from other Pleistocene sites in Sri Lanka e.g. Batadomba-lena [25]).

Phase C (Terminal Pleistocene) layers dip towards the cave wall–a pit was cut into these layers, and its fill is thus part of this phase. The deposits are heterogeneous mixtures of dark, organic-rich sandy loams and unconsolidated matrix-supported breccia with abundant charcoal and ash, either mixed with detrital sediment or present as distinct laminae/lenses. Notwithstanding its apparently short chronological range (13,000 to 12,000 cal. BP), Phase C contains the densest concentration of artefacts and human occupation debris in the Fa-Hien stratigraphy. Micromorphological analysis of basal Phase C deposits shows a very sharp-based accumulation of closely packed and imbricated wood charcoal and other charred biogenic particles (bone, snail shell, other plant remains [26]) in a matrix of fine grained charred organic debris, possibly a floor wash lag or a dump/rake out deposit. Overall, Phase C deposits probably resulted from a succession of erosion, colluviation and, perhaps, dumping episodes.

Phase B (early Holocene 8,700–8,000 cal. BP) deposits come above a sharp boundary that truncates the Terminal Pleistocene layers. Phase B comprises subhorizontal layers of light brown sandy silts, unconsolidated matrix-supported collapse breccia and ash accumulations, with a moderate amount of artefacts and habitation debris, interpreted as occupation deposits and floor wash colluvia. A large (diameter: 85cm, depth: 80cm), multi-stage pit was cut into the latter. This was filled with multiple layers of sandy silt, which may have resulted from colluviation, and ash and charcoal (much of this consists of burnt *Canarium* sp. seeds), possibly due to deliberate ash dumping [26]. Phase A (mid Holocene 6,000–4,000 cal. BP) begins with brown sandy loams and lenses of *Canarium* sp. seed charcoal (5,900 cal. BP), deposited directly above the Phase B pit and interpreted as *in situ* hearth deposits (probably from several burning episodes at the same spot), followed by colluvial deposition on the cave floor [26]. Above these come sharp-based, brown sandy and silty clays with little internal structure, interpreted as dumps, derived from prehistoric habitation contexts (*c*. 6,000 to 4,000 cal. BP). Brown sandy silts with gneiss spalls above Phase A (and immediately under the present cave floor) resulted from the recent extensive mining of the cave for fertiliser, and from ongoing colluviation [26].

Fa-Hien Lena Cave contains a large abundance and variety of organic materials. Monkeys and tree squirrels overwhelmingly dominate the faunal assemblage in all phases of site occupation, accounting for more than 70% of the identified remains [26]. Bone fragments with anthropogenic modification, ranging from burning to butchery marks, were recovered in all phases of site occupation. The assemblage also contains the earliest reported bone tool assemblage in South and Southeast Asia, and also one of the earliest beyond Africa [26]. Bone tools are found in all phases of site occupation [26]. The molluscan assemblage at Fa-Hien Cave is suggestive of a similar range and distribution of both freshwater and arboreal/terrestrial taxa occurring in all phases of site occupation, many taxa of which do not naturally occur in cave settings implying anthropogenic transport [26]. Furthermore, there is evidence for anthropogenic shell modification for aesthetic/ornamental purposes throughout the Late Pleistocene to mid-Holocene contexts. Utilised hematite and ochre are recorded in the all phases of the site and all phases of the site contain preserved remains of *Canarium* nuts and wild breadfruit [26].

## Materials and methods

The authors declare that the entire dataset is included within the Tables, Figures, and Supporting Information (S1 Table, S1–S3 Figs) included with this manuscript. The lithic material is located in the Department of Archaeology, Government of Sri Lanka, Colombo, Sri Lanka, in a permanent repository. While there are not specific accession numbers for each artefact, artefacts are stored under the site code ‘FH’ with clear contextual designators correlating to the phasing described here. All of the data reported in the paper are presented in the main text and Supporting Information.

The purpose of this study was to understand the nature and timing of technological behaviour in lithic assemblages from the Late Pleistocene to Holocene in Sri Lanka. Previous studies and reviews of Late Pleistocene/Holocene lithic materials from Sri Lanka document the discovery of microlithic tools [1,25,33,70], yet detailed analysis has been limited to a single study undertaken at Batadomba-lena, enabling comparisons with microlithic assemblages in India and South Africa [33]. A basic description of lithic material at Fa-Hien Lena was published in Wedage et al. [26] Supplementary Note 3 but, to date, no detailed systematic analysis of lithic production strategies, forms, and materials has been available. Here, we focus on the production processes, as well as raw materials and metrics, of Sri Lankan microliths and their *débitage*, from Late Pleistocene and Holocene contexts, thereby bringing them in line with descriptions of similar toolkits in other parts of the world [71–74]. All relevant analytical permits were obtained from the Department of Archaeology, Government of Sri Lanka for the work.

We employed a *chaîne opératoire* approach to investigate lithic reduction trajectories from stone tool assemblages recovered from Fa-Hien Lena Phases D to A, including all products and by-products of reduction. This approach employs a methodological framework that defines the reconstruction of the various processes of flake production–from the procurement of raw materials, through the phases of manufacture and utilization, to final discard [75–76]. Artefacts in each assemblage were split between raw material units (RMUs), defined according to stone macroscopic features including type of cortex, colour, grain size and texture [77]. Five basic categories of artefact were then identified: cores, flakes, chips (<10mm), retouched tools, and hammers. All cores and flakes were identified as either complete (Flake, Core) or broken (Flake Fragment, Core Fragment). The presence of cortex was recorded on all cores, flakes, and retouched tools, split into three categories of >50% cortex (referred to in the text as cortical), <50% cortex (referred to in the text as semi-cortical), and no cortex. Higher proportions of cortex remaining on an artefact are used to infer earlier stages of a reduction sequence.

Preliminary study [26] indicated the presence of a number of distinct artefact forms that we describe below and in Table 3. Two distinct reduction methods have been identified: freehand percussion and the bipolar knapping technique. Freehand percussion involves holding a core in the hand and striking it with a hammer to remove flakes, whereas in bipolar reduction, the core is held against an anvil when struck. The force applied from the hammerstone produces two opposed impact points: one on the upper face of the core and the second on the lower face that is in contact with the anvil. Since in this percussion technique (bipolar knapping *sensu stricto*) the core is perpendicular to the anvil, flakes are produced by the hitting of the hammerstone with the upper face and by the counterstrikes of the core with the anvil. During the analysis, the variant proposed by Hiscock [78] (bipolar-rested) has also been taken into consideration, in which the core morphology could change during the reduction and flakes could be produced without requiring two aligned opposing impact points. Generally, the morphology of the raw material determines how the pebble is placed on the anvil.

**Table 3:** Descriptions of key artefact types used in the analysis of lithic assemblages from Fa-Hien Lena.

Type	Description	References
Bipolar Core	Held against an anvil when struck with a hammer, bipolar cores often display two opposed and often crushed platforms for a single negative scar, yielding two distinct initiation points	[79–86]
Freehand Core	Held in the hand when struck, freehand cores exhibit a single initiation point for each negative scar	[79–86]
Core on Flake	A core produced on a flake (i.e. presenting a clear ventral surface) where negative scars appear targeted to remove flakes rather than impose shape	[79–86]
Microlith	Small flakes with a percussion axis length of less than 40mm that are targeted products of a reduction sequence	[44]
Bladelet	Elongate flakes (i.e., length:width >2:1), wider than they are thick, with less than 20% dorsal cortex, and exhibiting one or more dorsal ridges running roughly parallel to the percussion axis, with a percussion axis length of less than 40mm	[44]
Backed Microlith	Flakes or bladelets, with a percussion axis length of less than 40mm, whose lateral margins (usually one but sometimes two or more) have been partially or completely steeply retouched by using either bidirectional flaking or very steep and often stepped dorsal retouch	[44]
Bipolar Spall/ *bâtonnet*	Non-cortical flakes with longitudinal fractures and triangular/quadrangular sections	[87]
Splinter Flake	Splintered piece with more or less pronounced traces of longitudinal fracture	[87]

We have discriminated between the vertical axial knapping, when the pebble is oriented along the longer axis, and horizontal axial knapping, when the pebble is oriented along its shorter axis. During bipolar knapping, the striking angle tends to be ~90°, although some variations could be produced by fractures, changing of the striking platforms, or re-organization of the core volume []. The striking of the hammerstone on the upper face of the core may produce battering marks, Hertzian cone and linear striking platforms complemented by scaled or invasive bifacial detachments, or a pointed striking platform [79–82]. Analyses of bipolar reduction sequences were undertaken following more recent definitions based on experimental knapping data [79–82]. The bipolar technique differs from freehand knapping in terms of: a) fracture mechanics–the former includes wedging initiations, compression–propagation and preferential axial terminations; and, b) direction of the detachments–freehand reduction is often secant to one of the core axes and, when it is parallel, it requires the preparation of a striking platform [79–82]. Examination of core and flake scar patterning (e.g. unidirectional, bidirectional, orthogonal [perpendicular], radial) is used to infer patterns of core rotation [83–85].

The identification of the byproducts of bipolar percussion is not an easy task and it has been a matter of debate for decades [79–85]. The main issue is that the knapper has poor control over blanks produced, and flakes and fragments (e.g. basal, parasitic or irregular) could be detached from surfaces not involved in the knapping reduction. Generally, bipolar flakes show different morphologies and sizes, with diffuse bulbs of percussion, shattered platforms, and opposed fracture edges (e.g. hinge, step).

The use of excessive force while knapping or the presence of an irregular structure in a raw material can result in the production of flakes that are fragmented at the point of removal, rather than through their subsequent use or taphonomic impacts. In this investigation, flake fragments were differentiated following the criteria of Mourre [86]: a) siret *sensu strico* (x1) is considered a fracture parallel to the flaking axis that divides the blank in two parts, more or less equal; b) siret *sensu lato* (x2) is considered a fracture that removes a portion of the flake’s proximal side secant to the direction of the flaking axis; c) siret *sensu lato* (x3) is considered a fracture that removes two opposed portions of the flake’s proximal side obliquely to the direction of the flaking axis. Often, the remaining part of the platform shows a pointed morphology. Siret fractured flakes are typical byproducts of bipolar knapping strategies, though they may also be produced by freehand reduction methods. Backing, the use of steep or blunting retouch typically applied to the dorsal face of a flake, is a distinctive retouching strategy that is readily used to classify backed artefacts. The definition of microliths and microblades is more subjective. Here, we employ a 40mm cutoff to differentiate small from large flakes and blades, following the work by Petraglia and colleagues in southern India [44].

The maximum dimension and weights of all artefacts were recorded to evaluate metric characteristics of the lithic assemblages and enable assessment of targeted blank sizes, the impact of reduction intensity on flaking sequences, and discard thresholds. Statistical analysis of significant differences in maximum dimension and weight of cores and complete flakes was conducted using the free software PAST [88]. Preliminary testing using the Shapiro-Wilk test identified non-normal distributions of these data, resulting in the application of non-parametric tests (Mann Whitney test, α = 0.05) to examine differences in average artefact weight and maximum dimension between different raw materials and for cortical and non-cortical artefacts in each assemblage where n>20 (comparable to methods in Lewis [32]). Below this threshold descriptive statistics are used for comparisons. In order to compare the distribution of the length and weight of the cores, the data were firstly transformed to log10 values and then plotted in a scatterplot.

## Results

### Lithic analysis by Phase

The lithic assemblage of Fa-Hien Lena Cave is comprised of 9,216 artefacts distributed across four occupation phases (Table 4, S1 Table). The dominant raw material used at Fa-Hien Lena is quartz. River pebbles with smooth, cortical external surfaces were most likely gathered from the stream located 200m from the site (Fig 2). Macroscopic analysis permitted evaluation of the quality of quartz, ranging from higher quality varieties of automorphic quartz (crystal or hyaline, milky and rose) and poorer grade types of xenomorphic quartz (vein and grainy). A few chert flakes were also recognised, although there is no known source for this material within a 5km radius.

**Table 4:** Total number of lithic artefacts by chronological phases at Fa-Hien Lena Cave.

Layer	Flake	Bladelet	Flake Fragment	Chips	Tool	Core	Core Fragment	Hammer	Total
**A**	351	3	787	1144		22	16		2323
***%***	*15*.*1*	*0*.*1*	*33*.*9*	*49*.*2*	* *	*0*.*9*	*0*.*7*	* *	*100*
**B**	326	6	846	1262		20	10		2470
***%***	*13*.*2*	*0*.*2*	*34*.*3*	*51*.*1*	* *	*0*.*8*	*0*.*4*	* *	*100*
**C**	243	3	773	1639		18	13	4	2693
***%***	*9*	*0*.*1*	*28*.*7*	*60*.*9*	* *	*0*.*7*	*0*.*5*	*0*.*1*	*100*
**D**	290	13	659	743	3	9	9	4	1730
***%***	*16*.*8*	*0*.*8*	*38*.*1*	*42*.*9*	*0*.*2*	*0*.*5*	*0*.*5*	*0*.*2*	*100*
**Total**	1210	25	3065	4788	3	69	48	8	9216
***%***	*13*.*1*	*0*.*3*	*33*.*3*	*52*	*0*.*03*	*0*.*7*	*0*.*5*	*0*.*07*	*100*

#### Phase D (48,000–34,000 cal. BP)

**Fig 4:**
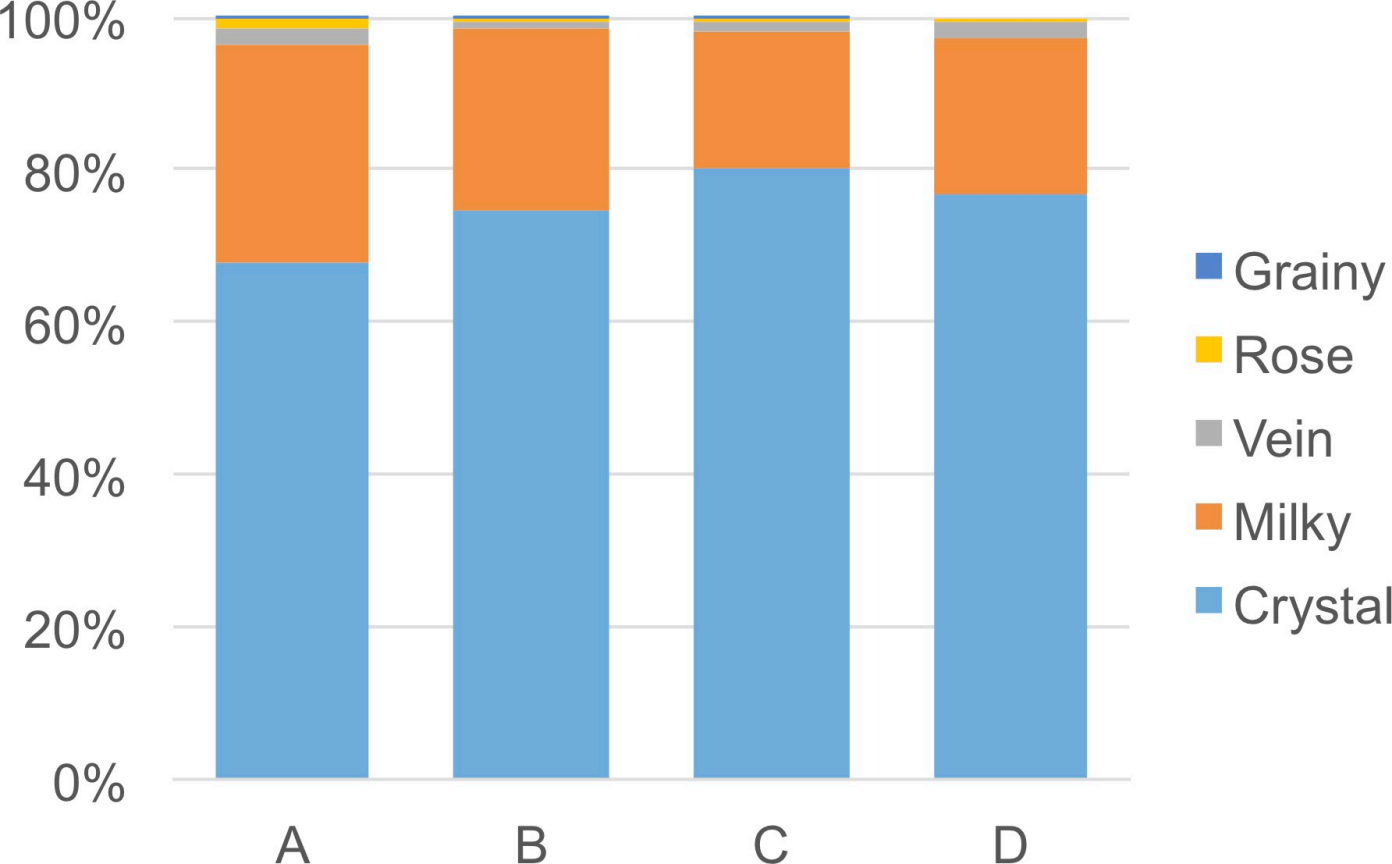
Frequency of quartz types by chronological phases recorded at Fa-Hien Lena Cave.

**Fig 5:**
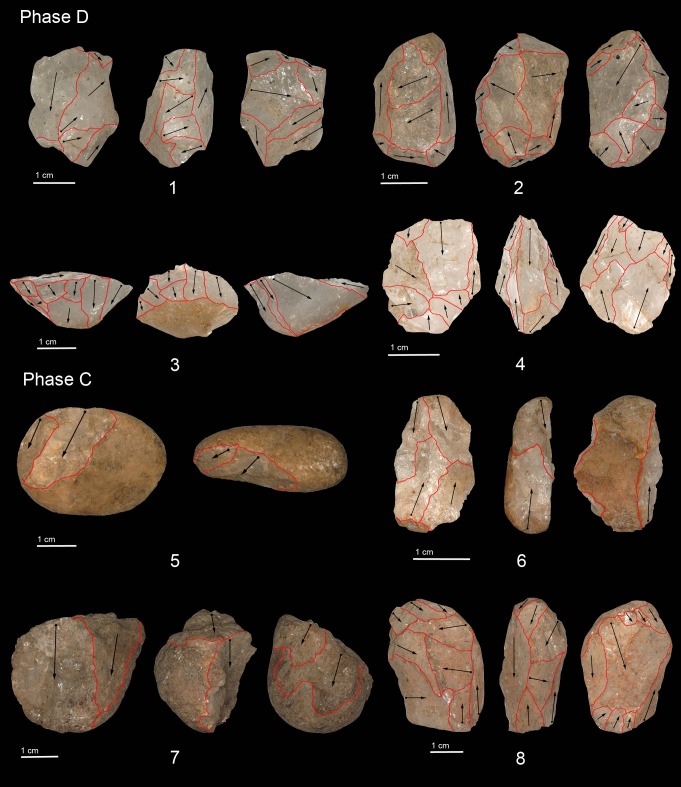
Cores of Phase D and C: bipolar core (no. 4, 5, 6), bipolar orthogonal core (no. 1, 2, 7, 8), unidirectional abrupt core (no. 3).

The lithic assemblage of Phase D is composed of 1,730 artefacts (1,726 débitage byproducts and four hammerstones). The assemblage is dominated by small chips (43%) and flake fragments (38%), with complete flakes and cores occurring in smaller amounts (Tables 4 and 5). Crystal and milky quartz is the primary raw material (Table 4, Fig 4). Technological analysis of the core assemblage revealed the use of the bipolar method (Fig 5: 1, 2, 4–8). Four cores in crystal quartz were reduced along their longest axis (vertical axial). The flaking surfaces are characterised by bidirectional detachments produced by the hammerstones and contact with the anvil. In two examples, the repeated battering of the hammerstones produced bifacial detachments on the proximal sides creating platforms with conical morphologies (Fig 5: 4). The use of a freehand knapping strategy is documented in two exhausted core-on-flakes. In the first example, the ventral surface of a flake was used as the striking platform for the abrupt unidirectional production of elongated flakes (Fig 5: 3). In the second core, broken during the knapping event, two small flakes were detached from the ventral surface.

**Table 5:** Counts, mean (μ) and standard deviation (σ) of the metric attribute (mm) of the cores in crystal and milky quartz of Fa-Hien Lena Cave.

Phase	**Attributes**	Crystal quartz			Milky quartz		
		N°	μ	σ	N°	μ	σ
**A**	Length	6	23.7	*9*.*2*	14	28.4	*8*.*04*
	Width		18.7	*5*.*2*		28.5	*11*.*2*
	Thickness		13.4	*4*.*7*		18.9	*7*.*05*
**B**	Length	8	21.9	*5*.*4*	12	35.2	*14*.*3*
	Width		21.9	*5*.*3*		30.9	*12*.*9*
	Thickness		15.6	*6*.*6*		21.1	*6*.*5*
**C**	Length	7	23.5	*10*.*9*	10	29.8	*6*.*03*
	Width		16.9	*8*.*5*		24.8	*8*.*9*
	Thickness		12.4	*5*.*2*		17.5	*6*.*9*
**D**	Length	5	26.4	*4*.*3*	4	30.8	*5*.*2*
	Width		22.2	*4*.*6*		25.6	*4*.*05*
	Thickness		14.6	*2*.*6*		21.7	*2*.*3*

The analysis of the bipolar cores in milky quartz reveals a different approach to the knapping process. Generally, the flaking sequence started with recurrent detachments along the longest axis, yielding by-products with rectilinear or convex edges. One core was discarded after having been fractured at the distal end. The remaining three cores at some point were rotated by 90° and a new production on the shorter side (horizontal axial) was initiated (Fig 5: 1, 2). Rotation of the cores, with simultaneous changes of the striking platforms, is documented several times and, most likely, was performed in order to facilitate a more secure placing of the core on the anvil as the morphology of the pebble changed during the knapping process. This technical expediency produced orthogonal scars on the ventral surfaces of the cores. Measurement of the size of the scar negatives indicated that the produced blanks would have been small, generally shorter than 20mm.

A comparison of the descriptive statistics suggests comparable average lengths and widths for crystal and milky quartz cores, but the milky quartz cores are notably thicker on average (Table 5). The higher quality of the crystal quartz probably allowed for better exploitation of the pebbles, whereas milky quartz cores were reduced less intensely. This result is also supported by the higher frequency of crystal quartz complete flakes in comparison with the other types of quartz (Table 6).

**Table 6:** Total number and percentage of lithic artefacts in Phase D.

	Crystal	*%*	Milky	*%*	Vein	*%*	Rose	*%*	Total	*%*
**Cortical flake >50%**	11	*0*.*8*	5	*1*.*4*	1	*3*.*1*		* *	17	*1*
**Cortical flake <50%**	29	*2*.*2*	11	*3*.*1*	3	*9*.*4*	2	*16*.*7*	45	*2*.*6*
**Cortical core-edge flake**	2	*0*.*2*		* *		* *		* *	2	*0*.*1*
**Flake**	175	*13*.*2*	27	*7*.*6*	5	*15*.*6*		* *	207	*12*
**Splinter flake**	16	*1*.*2*	1	*0*.*3*		* *		* *	17	*1*
**Core-edge flake**	1	*0*.*1*	1	*0*.*3*		* *		* *	2	*0*.*1*
**Bladelet**	13	*1*		* *		* *		* *	13	*0*.*8*
**Bâtonnet**	6	*0*.*5*	1	*0*.*3*		* *		* *	7	*0*.*4*
**Cortical flake fragment**	34	*2*.*6*	16	*4*.*5*	2	*6*.*3*	5	*41*.*7*	57	*3*.*3*
**Siret x1**	9	*0*.*7*	4	*1*.*1*		* *		* *	13	*0*.*8*
**Siret x2**	4	*0*.*3*		* *		* *		* *	4	*0*.*2*
**Siret x3**	4	*0*.*3*	1	*0*.*3*	1	*3*.*1*	1	*8*.*3*	7	*0*.*4*
**Flake fragment**	320	*24*.*1*	92	*26*	11	*34*.*4*	3	*25*	426	*24*.*7*
**Siret x1**	53	*4*	14	*4*	2	*6*.*3*		* *	69	*4*
**Siret x2**	35	*2*.*6*	6	*1*.*7*		* *		* *	41	*2*.*4*
**Siret x3**	31	*2*.*3*	4	*1*.*1*		* *		* *	35	*2*
**Chips**	574	*43*.*2*	163	*46*	6	*18*.*8*		* *	743	*43*
**Microlith**	3	*0*.*2*		* *		* *		* *	3	*0*.*2*
**Core**	5	*0*.*4*	4	*1*.*1*		* *		* *	9	*0*.*5*
**Core fragment**	3	*0*.*2*	4	*1*.*1*	1	*3*.*1*	1	*8*.*3*	9	*0*.*5*
**Total**	1328	*100*	354	*100*	32	*100*	12	*100*	1726	*100*

**Fig 6:**
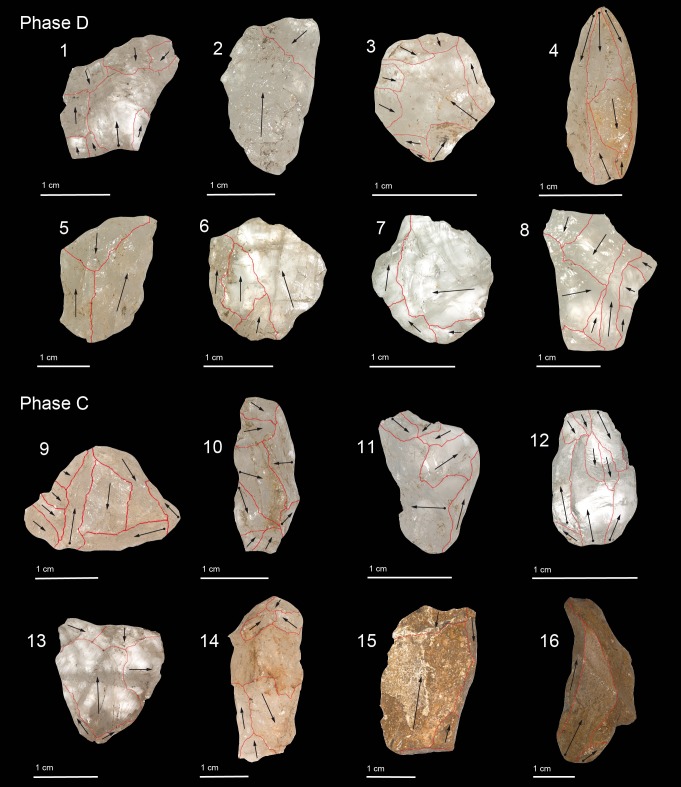
Flakes of Phase D and C: bipolar flake (no. 1, 5, 6, 14), bipolar orthogonal flake (no. 3, 7, 8, 9, 11, 13), splinter flake (no. 4, 10, 12), bipolar flake with siret sensu stricto fracture (no. 2), unidirectional flake fragment on chert (no. 15), semi-cortical flake on chert (no. 16).

The analysis of the crystal and milky quartz flake assemblages indicates that primary decortification and tool production occurred on site, including the presence of core edge flakes that may have helped to manage flaking surfaces (Table 6, Fig 6). Conversely, artefacts in vein and rose quartz are very few and mostly associated with the early stages of reduction (Table 6). The data on the crystal quartz reveals higher frequencies of cortical blanks and unbroken flakes and bladelets (Table 6). No clear evidence for the application of a dedicated laminar reduction approach is evident, suggesting that these bladelets were the product of bipolar reduction. Although the rotation of the cores is documented only in milky quartz, orthogonal scars were found in eleven flakes of crystal quartz (Fig 6: 7) and in two flakes of milky quartz (Fig 6: 3, 8). The statistical comparison between the dimensions of unbroken blanks indicates a significant difference between the average length value of cortical (n = 42) and non-cortical (n = 205) flakes in crystal quartz (Mann Whitney *p* = 0.0012). Descriptive statistics suggest no substantive difference between cortical (n = 16) and non-cortical (n = 29) flakes in milky quartz (Tables 7 and 8).

**Table 7:** Count, mean (μ) and standard deviation (σ) of the length (mm) of cortical flakes in Fa-Hien Lena Cave.

Phase	Crystal			Milky			Vein			Rose			Grainy			Chert	
	N°	μ	σ	N°	μ	σ	N°	μ	σ	N°	μ	σ	N°	μ	σ	N°	μ
**A**	60	18.3	*6*.*2*	57	21.4	*7*.*3*	4	20.2	*10*.*1*	6	19.4	*3*.*9*	2	42.5	*15*.*3*		
**B**	47	18.9	*5*.*9*	47	22.4	*10*.*5*	5	22.2	*2*.*8*	3	29.4	*2*.*5*	1	70.7	* *		
**C**	44	19.2	*7*.*2*	22	21.6	*8*.*4*	2	17.8	*1*.*5*	5	25.8	*8*.*1*			* *	1	58.1
**D**	42	20	*7*.*01*	16	22.5	*6*.*8*	4	19.5	*3*.*7*	2	12.8	*2*.*5*			* *		

**Table 8:** Count, mean (μ) and standard deviation (σ) of the length (mm) of complete flakes and bladelets in Fa-Hien Lena Cave.

Phase	Crystal			Milky			Vein			Rose			Grainy			Chert	
	N°	μ	σ	N°	μ	σ	N°	μ	σ	N°	μ	σ	N°	μ	σ	N°	μ
**A**	60	18.3	*6*.*2*	57	21.4	*7*.*3*	4	20.2	*10*.*1*	6	19.4	*3*.*9*	2	42.5	*15*.*3*		
**B**	47	18.9	*5*.*9*	47	22.4	*10*.*5*	5	22.2	*2*.*8*	3	29.4	*2*.*5*	1	70.7	* *		
**C**	44	19.2	*7*.*2*	22	21.6	*8*.*4*	2	17.8	*1*.*5*	5	25.8	*8*.*1*			* *	1	58.1
**D**	42	20	*7*.*01*	16	22.5	*6*.*8*	4	19.5	*3*.*7*	2	12.8	*2*.*5*			* *		

Typical by-products of bipolar reduction, including *bâtonnet* flakes and splinter flakes [44,89], are present in the assemblage (Table 6). Splinter flakes could be produced at any stage of the knapping process and were identified in seven semi-cortical flakes of crystal quartz and one semi-cortical flake of milky quartz (Fig 6: 4). Since quartz is not a homogeneous raw material, the presence of internal flaws and crystalline surfaces could cause unintentional breakages during knapping events [90]. Due to the crystallographic features of the quartz pebbles, a high percentage of fragments are recorded within the assemblage. Within this category, siret fractures *sensu stricto* are common (Fig 6: 2) whereas siret breakages *sensu lato* are recognised in lesser frequencies (Table 6).

**Fig 7:**
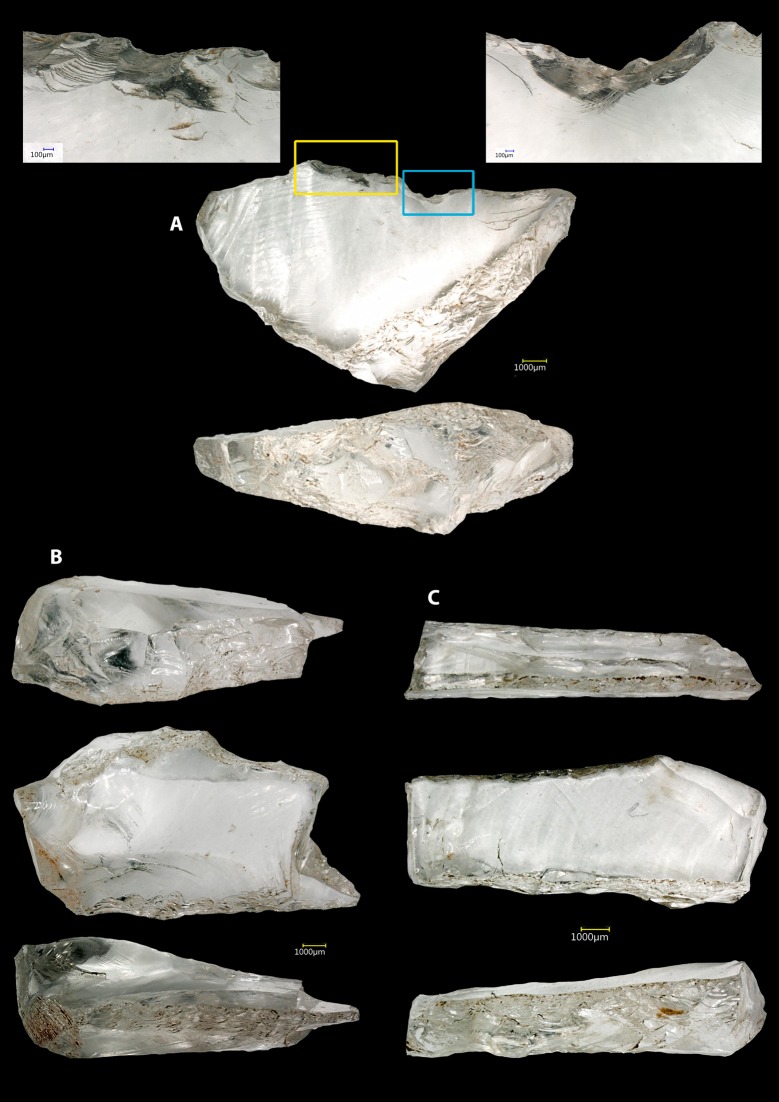
Microlithic tools in Phase D of Fa-Hien Lena Cave.

Retouched tools occur in the form of three microliths in crystal quartz (Fig 7): a) a crescent microlith with a continuous backed retouch along one side and two notch fractures on the cutting edge found in layer 165; b) a laminar microlith fragment with a bend fracture and a backed retouch on both edges found in layer 158; and, c) a laminar microlith with a step terminating breakage and a backed retouch on both edges found in layer 175. Ongoing functional study of these artefacts by Dr. Michelle Langley, Griffith University, Brisbane, Australia is examining whether the breaks that have been identified relate to their use (e.g. as impact fractures).

#### Phase C (13,000–12,000 cal. BP)

The lithic assemblage of Phase C comprises 2,693 artefacts (2,689 lithic items and four hammerstones) (Tables 4 and 9). Small chips and flake fragments account for 89.6% of the lithic materials whereas complete flakes and cores are recorded in a much smaller number (Table 6). Crystal and milky quartz are the most commonly used raw materials followed by vein, rose, and grainy quartz (Table 9, Fig 4). The diacritic reading of the scars on cores and flakes allowed for the identification of the use of bipolar knapping on the anvil (Fig 5: 5–8). Cores in crystal quartz are small and generally exploited along their longest axis for short knapping sequences. In one pebble, the reduction was horizontal axial and the core was discarded after a short knapping sequence (Fig 5: 5). The negative scars on previous detachments are small and less than 15mm. Only one artefact shows rotation of the striking platform from a vertical to horizontal reduction (Fig 5: 8). This latter core is also covered in red ochre.

**Table 9:** Total number and percentage of lithic artefacts in Phase C.

	Crystal	*%*	Milky	*%*	Vein	*%*	Rose	*%*	Grainy	*%*	Chert	*%*	Total	*%*
**Cortical flake >50%**	6	*0*.*3*	5	*1*	1	*3*.*3*	3	*25*		* *		* *	15	*0*.*6*
**Cortical flake <50%**	33	*1*.*5*	14	*2*.*9*	1	*3*.*3*	2	*16*.*7*		* *	1	*25*	51	*1*.*9*
**Cortical core-edge flake**	5	*0*.*2*	3	*0*.*6*		* *		* *		* *		* *	8	*0*.*3*
**Flake**	129	*6*	19	*3*.*9*	3	*10*		* *		* *	2	*50*	153	*5*.*7*
**Splinter flake**	7	*0*.*3*	5	*1*		* *		* *		* *		* *	12	*0*.*4*
**Core-edge flake**	3	*0*.*1*	1	*0*.*2*		* *		* *		* *		* *	4	*0*.*1*
**Bladelet**	3	*0*.*1*		* *		* *		* *		* *		* *	3	*0*.*1*
**Bâtonnet**	3	*0*.*1*		* *		* *		* *		* *		* *	3	*0*.*1*
**Cortical flake fragment**	45	*2*.*1*	36	*7*.*4*	6	*20*	1	*8*.*3*	2	*28*.*6*		* *	90	*3*.*3*
**Siret x1**	7	*0*.*3*		* *	1	*3*.*3*	2	*16*.*7*		* *		* *	10	*0*.*4*
**Siret x2**	4	*0*.*2*		* *		* *		* *	1	*14*.*3*		* *	5	*0*.*2*
**Siret x3**	1	*0*.*0*		* *		* *		* *		* *		* *	1	*0*.*0*
**Flake fragment**	391	*18*.*2*	121	*24*.*9*	12	*40*	2	*16*.*7*	4	*57*.*1*	1	*25*	531	*19*.*7*
**Siret x1**	65	*3*	19	*3*.*9*	1	*3*.*3*		* *		* *		* *	85	*3*.*2*
**Siret x2**	19	*0*.*9*	3	*0*.*6*		* *		* *		* *		* *	22	*0*.*8*
**Siret x3**	20	*0*.*9*	5	*1*	1	*3*.*3*		* *		* *		* *	26	*1*
**Chips**	1393	*64*.*8*	241	*49*.*7*	4	*13*.*3*	1	*8*.*3*		* *		* *	1639	*61*
**Core**	7	*0*.*3*	10	*2*.*1*		* *	1	*8*.*3*		* *		* *	18	*0*.*7*
**Core fragment**	10	*0*.*5*	3	*0*.*6*		* *		* *		* *		* *	13	*0*.*5*
**Total**	2151	*100*	485	*100*	30	*100*	12	*100*	7	*100*	4	*100*	2689	*100*

In milky quartz, seven cores are exploited vertically but, in some of them, internal flaws caused unexpected breakages during knapping. In two artefacts, the battering of the hammerstone caused a longitudinal fracture, removing distal portions of the core (Fig 5: 6). In another, the blows on the anvil provoked the fracture of the distal margin. A fifth core was discarded because impurities in the quartz pebble caused detachment of two large portions of the flaking surface and created a step on the mesial side. Horizontal reduction of the flaking surface is documented in four of the remaining cores (Fig 5: 7). These cores are small, and their rotation could be interpreted as technical expediency to exploit the raw material more efficiently. A rose quartz core was also discovered in the assemblage (22.2 x 26.6 x 16.2mm). This artefact is small with a pronounced Hertzian cone on the proximal side. The flaking surface shows bidirectional negative scars and a fracture on the distal margin. In contrast to Phase D, average descriptive statistics suggest no notable differences in core sizes in Phase C (Table 5).

In the flake assemblage complete technological sequences are documented in crystal and milky quartz, whereas in the other quartz types only a few flakes were produced during phases of decortication (Table 9, Fig 6). Most of the cores retain cortical portions on their surfaces. The recovery of several cortical flakes supports the hypothesis that knapping activities were carried out at the site. A significant statistical difference is recorded between the average length value of unbroken cortical (n = 44) and non-cortical (n = 142) flakes in crystal quartz (Mann Whitney *p =* 0.0002), whereas no difference is documented between cortical (n = 22) and non-cortical (n = 25) flakes in milky quartz (Mann Whitney *p* = 0.4620) (Tables 7 and 8).

The data suggests that quartz pebbles were of low quality, since most the lithic items broke during knapping events (Table 9). Within fragments, siret fracture *sensu strico* are common (3.6%), while siret fracture *sensu latu* are recorded in lesser frequencies (Table 9). Splinter flakes were noted in several unbroken blanks (Fig 6: 10, 12), six semi-cortical flakes in crystal quartz, in one cortical flake, and three semi-cortical flakes in milky quartz (Table 9). A few bladelets are also present, although it appears that their production was random and not planned as in other technologies. In the complete flake assemblage, orthogonal scars on the dorsal surface attest to the rotation of the striking platform in three crystal quartz artefacts (Fig 6: 9, 11) and in one flake on milky quartz.

The Phase C assemblage also included four chert artefacts: a semi-cortical flake with a cortical platform and a unidirectional negative scar on the dorsal surface (Fig 6: 16), two small unidirectional flakes (Fig 6: 15), and a proximal fragment (Table 9). These flakes were produced from different chert pebbles and they were introduced to the site as transported artefacts. The technological analysis of the flakes indicates that they were produced using the unidirectional method and not the bipolar method.

#### Phase B (8,700–8,000 cal. BP)

**Fig 8:**
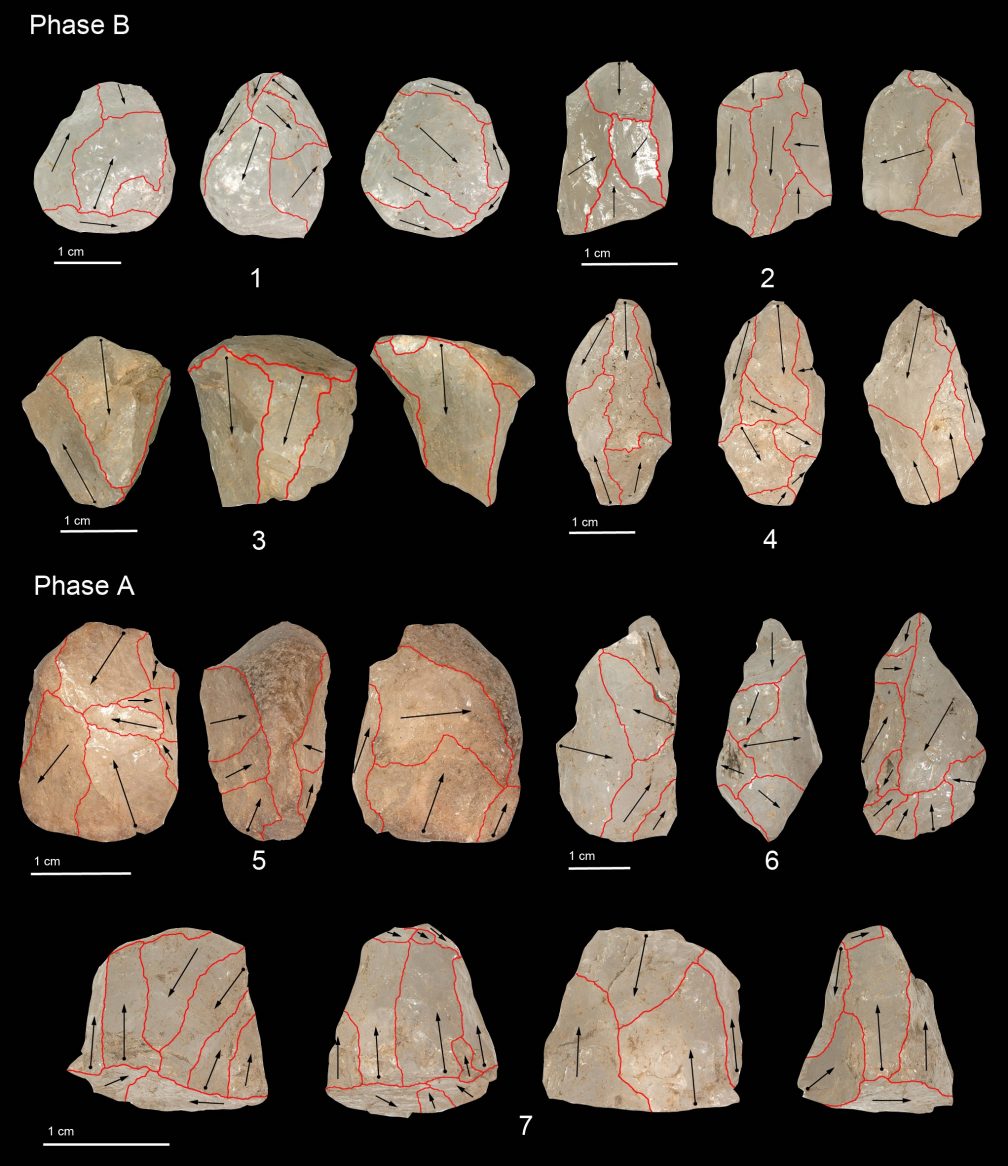
Cores in Phase B and A: bipolar core (3, 7), bipolar orthogonal core (1, 2, 4, 5, 6).

The lithic assemblage of Phase B is composed of 2,470 items, predominantly small chips and flake fragments (85.4%) (Table 4). The raw materials used mostly are crystal and milky quartz (Table 10, Fig 4). Technological analysis of the core and flake collection reveals the use of the bipolar method (Fig 8). In three cores made from crystal quartz, the pebbles are reduced along the longest axis. In another, the fractures on the striking platform shaped the blank into a pyramidal morphology. In the other five cores, the knappers changed the striking platforms, rotating the artefact flaking axis by 90° (Fig 8: 1, 2). It is worth noting that the striking platforms of these samples are more crushed in comparison with those of the previous phases, probably due to the use of heavier hammerstones. In one core, vertical exploitation resulted in a fracture, leading to the use of a new striking platform.

**Table 10:** Total number and percentage of lithic artefacts in Phase B.

	Crystal	*%*	Milky	*%*	Vein	*%*	Rose	*%*	Grainy	*%*	Chert	*%*	Total	*%*
**Cortical flake >50%**	15	*0*.*8*	15	*2*.*6*	2	*9*.*1*	1	*9*.*1*	1	*14*.*3*	1	*33*.*3*	35	*1*.*4*
**Cortical flake <50%**	32	*1*.*7*	29	*5*	2	*9*.*1*	2	*18*.*2*		* *		* *	65	*2*.*6*
**Cortical core-edge flake**		* *	3	*0*.*5*	1	*4*.*5*		* *		* *		* *	4	*0*.*2*
**Flake**	149	*8*.*1*	43	*7*.*4*	3	*13*.*6*		* *	2	*28*.*6*	1	*33*.*3*	198	*8*
**Splinter flake**	15	*0*.*8*	9	*1*.*5*		* *		* *		* *		* *	24	*1*
**Bladelet**	5	*0*.*3*	1	*0*.*2*		* *		* *		* *		* *	6	*0*.*2*
**Bâtonnet**	2	*0*.*1*		* *		* *		* *		* *		* *	2	*0*.*1*
**Cortical flake fragment**	53	*2*.*9*	36	*6*.*2*	3	*13*.*6*	2	*18*.*2*		* *		* *	94	*3*.*8*
**Siret x1**	5	*0*.*3*	7	*1*.*2*	1	*4*.*5*	1	*9*.*1*		* *		* *	14	*0*.*6*
**Siret x2**	3	*0*.*2*	2	*0*.*3*		* *		* *		* *		* *	5	*0*.*2*
**Siret x3**	3	*0*.*2*	5	*0*.*9*		* *		* *		* *		* *	8	*0*.*3*
**Flake fragment**	434	*23*.*5*	144	*24*.*7*	2	*9*.*1*	3	*27*.*3*	1	*14*.*3*	1	*33*.*3*	585	*23*.*7*
**Siret x1**	52	*2*.*8*	34	*5*.*8*	1	*4*.*5*	1	*9*.*1*		* *		* *	88	*3*.*6*
**Siret x2**	22	*1*.*2*	6	*1*		* *		* *		* *		* *	28	*1*.*1*
**Siret x3**	17	*0*.*9*	5	*0*.*9*		* *		* *		* *		* *	22	*0*.*9*
**Chips**	1027	*55*.*7*	224	*38*.*4*	7	*31*.*8*	1	*9*.*1*	3	*42*.*9*		* *	1262	*51*.*1*
**Core**	8	*0*.*4*	12	*2*.*1*		* *		* *		* *		* *	20	*0*.*8*
**Core fragment**	1	*0*.*1*	9	*1*.*5*		* *		* *		* *		* *	10	*0*.*4*
**Total**	1843	*100*	584	*100*	22	*100*	11	*100*	7	*100*	3	*100*	2470	*100*

In the milky quartz assemblage, eight cores were exploited using vertical reduction (Fig 8: 3) and two of them display a pronounced Hertzian cone on the proximal side, probably caused by the recurrent battering on the same area of the platform. In five cores, the internal flaws on the raw material resulted in fracture of the striking platforms. Within this group, three artefacts document recurrent production even after breakage, with cores being shaped into a pyramidal morphology where the pointed edge is the platform and the flat surface is the distal side. A total of four cores show changes in the direction of the reduction from vertical to horizontal with a rotation of the artefacts by 90° (Fig 8: 4). On one artefact, the change in the striking platforms is likely related to the more efficient exploitation of the core since, during reduction, different fractures limited the amount of raw material available. Descriptive statistics for crystal and milky quartz indicate substantial differences in size, with milky quartz cores typically larger across all three dimensions (Table 5).

**Fig 9:**
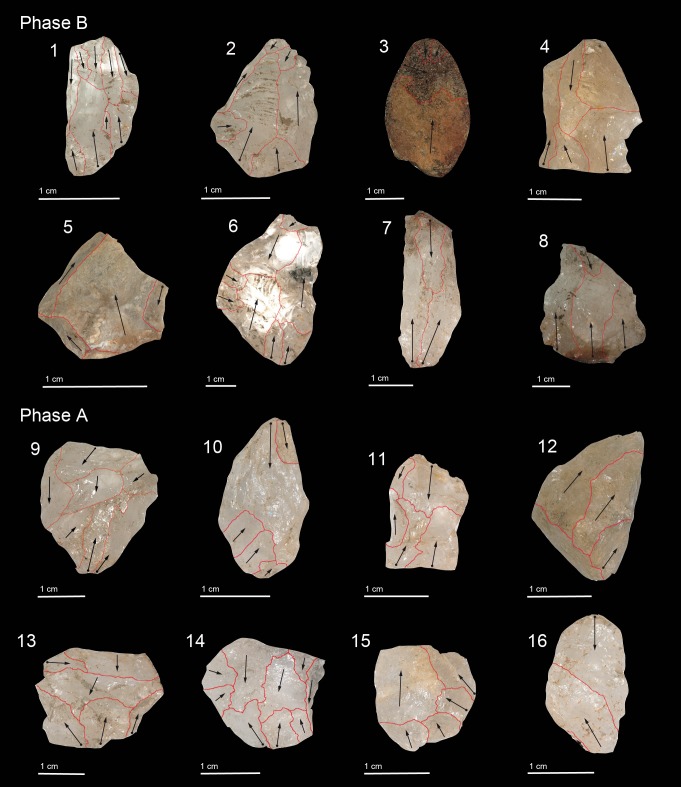
Flakes in Phase B and A: bipolar flake (1, 2, 3, 8, 12, 15, 16), bipolar orthogonal flake (9), splinter flake (4, 6, 7, 10, 11, 13, 14), unidirectional flake fragment in chert (5).

In the flake assemblage (Fig 9), complete knapping sequences are recorded in crystal and milky quartz. By contrast, in the other quartz types pebble decortication is primarily documented (Table 10). Although 65% of the cores retain some cortex on their surfaces, the amount of cortical items is larger than in the assemblages from the previous Phases (Table 10). Within the group of complete flakes, only two items in crystal quartz and one blank in milky quartz show orthogonal detachments produced during the rotation of the striking platforms. Comparison between unbroken cortical (n = 47) and non-cortical (n = 169) crystal quartz flakes indicates significant difference in the average length (Mann Whitney *p* = < 0.0001), but not between cortical (n = 47) and non-cortical (n = 53) milky quartz flakes (Mann Whitney *p* = 0.5297) (Tables 7 and 8).

Bladelets and *bâtonnets* are limited in the assemblage, whereas splinter flakes are found in four semi-cortical flakes in crystal quartz (Fig 9: 4, 6), two cortical flakes in milk quartz, four semi-cortical flakes in milky quartz (Fig 9: 7), and in one semi-cortical blank in vein quartz. Broken artefacts are very common in the assemblage and siret fractures *sensu strico* are more frequent than the other types of siret *sensu lato* in cortical and non-cortical fragments. In the assemblage from Phase B, three artefacts made from chert pebbles are also documented: one cortical flake, one proximal fragment (Fig 9: 5), and one unidirectional flake (Table 10). The chert RMU’s are different from those recorded in Phase C, making it difficult to assess if they were collected in the same area.

#### Phase A (6,000–4,000 cal. BP)

The lithic assemblage of Phase A comprises 2,323 artefacts and includes mostly small chips (49.2%) and flake fragments (33.9%) (Table 4). The predominant raw materials are crystal and milky quartz, with vein, rose, and grainy quartz being less frequent (Table 11, Fig 4). Technological analysis of the core and flake assemblage points to the use of the bipolar method (Fig 8). Four cores in crystal quartz were exploited along their longest axis and, in the case of two artefacts, internal flaws caused fracture of the striking platforms (Fig 8: 7). In addition, two other cores show that the striking platforms were rotated by 90° and the lateral plain surfaces were used for short, horizontal reduction sequences (Fig 8: 5, 6).

**Table 11:** Total number and percentage of lithic artefacts in Phase A.

	Crystal	*%*	Milk	*%*	Vein	*%*	Rose	*%*	Grain	*%*	Chert	*%*	Total	*%*
**Cortical flake >50%**	13	*0*.*8*	23	*3*.*5*	2	*4*.*3*	2	*6*.*3*	2	*50*		* *	42	*1*.*8*
**Cortical flake <50%**	46	*2*.*9*	34	*5*.*1*	2	*4*.*3*	4	*12*.*5*		* *		* *	86	*3*.*7*
**Cortical core-edge flake**	1	*0*.*1*				* *		* *		* *		* *	1	*0*.*0*
**Flake**	133	*8*.*4*	49	*7*.*4*	4	*8*.*7*		* *		* *		* *	186	*8*
**Splinter flake**	16	*1*	18	*2*.*7*		* *		* *		* *		* *	34	*1*.*5*
**Core-edge flake**	2	*0*.*1*				* *		* *		* *		* *	2	*0*.*1*
**Bladelet**	2	*0*.*1*	1	*0*.*2*		* *		* *		* *		* *	3	*0*.*1*
**Bâtonnet**	3	*0*.*2*				* *		* *		* *		* *	3	*0*.*1*
**Cortical flake fragment**	57	*3*.*6*	77	*11*.*6*	11	*23*.*9*	9	*28*.*1*		* *		* *	154	*6*.*6*
**Siret x1**	5	*0*.*3*	13	*2*	1	*2*.*2*	1	*3*.*1*		* *		* *	20	*0*.*9*
**Siret x2**	4	*0*.*3*	5	*0*.*8*		* *		* *		* *		* *	9	*0*.*4*
**Siret x3**	5	*0*.*3*	3	*0*.*5*		* *		* *		* *		* *	8	*0*.*3*
**Flake fragment**	263	*16*.*7*	176	*26*.*5*	9	*19*.*6*	5	*15*.*6*	1	*25*		* *	454	*19*.*5*
**Siret x1**	66	*4*.*2*	35	*5*.*3*	2	*4*.*3*		* *		* *	1	*100*	104	*4*.*5*
**Siret x2**	12	*0*.*8*	4	*0*.*6*		* *		* *		* *		* *	16	*0*.*7*
**Siret x3**	12	*0*.*8*	7	*1*.*1*		* *		* *		* *		* *	19	*0*.*8*
**Chips**	923	*58*.*6*	195	*29*.*4*	14	*30*.*4*	11	*34*.*4*	1	*25*		* *	1144	*49*.*2*
**Core**	6	*0*.*4*	15	*2*.*3*	1	*2*.*2*		* *		* *		* *	22	*0*.*9*
**Core fragment**	7	*0*.*4*	9	*1*.*4*		* *		* *		* *		* *	16	*0*.*7*
**Total**	1576	*100*	664	*100*	46	*100*	32	*100*	4	*100*	1	*100*	2323	*100*

In the milky quartz assemblage vertical reduction of the core volume is documented in eight cores, while the morphology of the pebbles favoured horizontal exploitation in two cores. In three artefacts the hammerstones broke the striking platforms, shaping the proximal surface into a conical shape. Short knapping sequences are recognised in three cores, although the pebbles retain more volume for exploitation. The change in the direction of the flaking reduction from vertical to horizontal is recorded on five cores; on two of them a fracture was used as a second striking platform. The dimensions of the core byproducts are shorter than 20mm. Despite excluding a milky quartz core weighing 457.8g as an outlier, descriptive statistics for the cores suggest milky quartz cores are notably larger than crystal quartz cores across all three dimensions (Table 5). A core in vein quartz with a cortical portion on the dorsal surface was also found in the assemblage (24.9 x 17.2 x 10.7 mm). During reduction, the core was rotated for better exploitation of the volume, producing flakes smaller than 17mm.

In the flake assemblage, different stages of core reduction, from decortication to production, are recorded in the crystal and milky quartz assemblages. By contrast to the other quartz types, most of the artefacts are associated with the cortical categories (Table 11). Comparison between the length of complete cortical (n = 57) and non-cortical (n = 68) milky quartz flakes indicates similar average values (Mann Whitney *p* = 0.3203) whereas a significant statistical difference is recorded between cortical (n = 60) and non-cortical (n = 153) crystal quartz flakes (Mann Whitney *p* = 0.0006) (Tables 7 and 8). Splinter flakes are more common in Phase A than other phases (Table 11, Fig 9: 10, 11, 13, 14). They were identified in six cortical and five semi-cortical flakes in crystal quartz, six cortical and six semi-cortical in milky quartz, one cortical and two semi-cortical flakes in rose quartz, and in one semi-cortical blank in vein quartz. The number of bladelets and *bâtonnets* is very small, while, for complete flakes, orthogonal scar negatives are documented only in one flake in crystal quartz (Fig 9: 9). Fragments are abundant in the assemblage and siret fractures *sensu strico* are more frequent than siret fractures *sensu lato* (Table 11). Siret breakage was also identified in the single chert artefact discovered in the assemblage.

#### Fa-Hien Lena lithic technology through time

The Fa-Hien Lena lithic assemblage reveals remarkable long-term technological continuity in the Wet Zone rainforest of Sri Lanka, from the Late Pleistocene into the Holocene. The earliest occupation of this rainforest environment, *c*. 48,000–45,000 cal. years BP, is in part represented by the exploitation of local quartz pebbles using the bipolar method. Although there is a *c*. 20,000-year hiatus in the stratigraphic sequence between Phases D and C, the technological approaches in all phases are similar, consisting of the exploitation of the longest axis of cores and the rotation of striking platforms. Raw material provisioning was generally local: quartz pebbles were apparently collected from a nearby stream through all phases of site occupation. The diachronic comparison of raw materials indicates a uniform preference of crystal quartz, whereas a slight increase in the use of milky quartz pebbles is documented during the Early and Mid-Holocene (Fig 4). A few chert flakes of different RMUs were also recognised in the assemblage. Since sedimentary bedrock formations are not reported in the area, these chert artefacts were probably transported to the site as part of the toolkit, but the location of the chert source(s) is still unknown. Further work on raw material procurement will hopefully shed light on the mobility patterns of prehistoric foragers at Fa-Hien Lena Cave.

**Fig 10:**
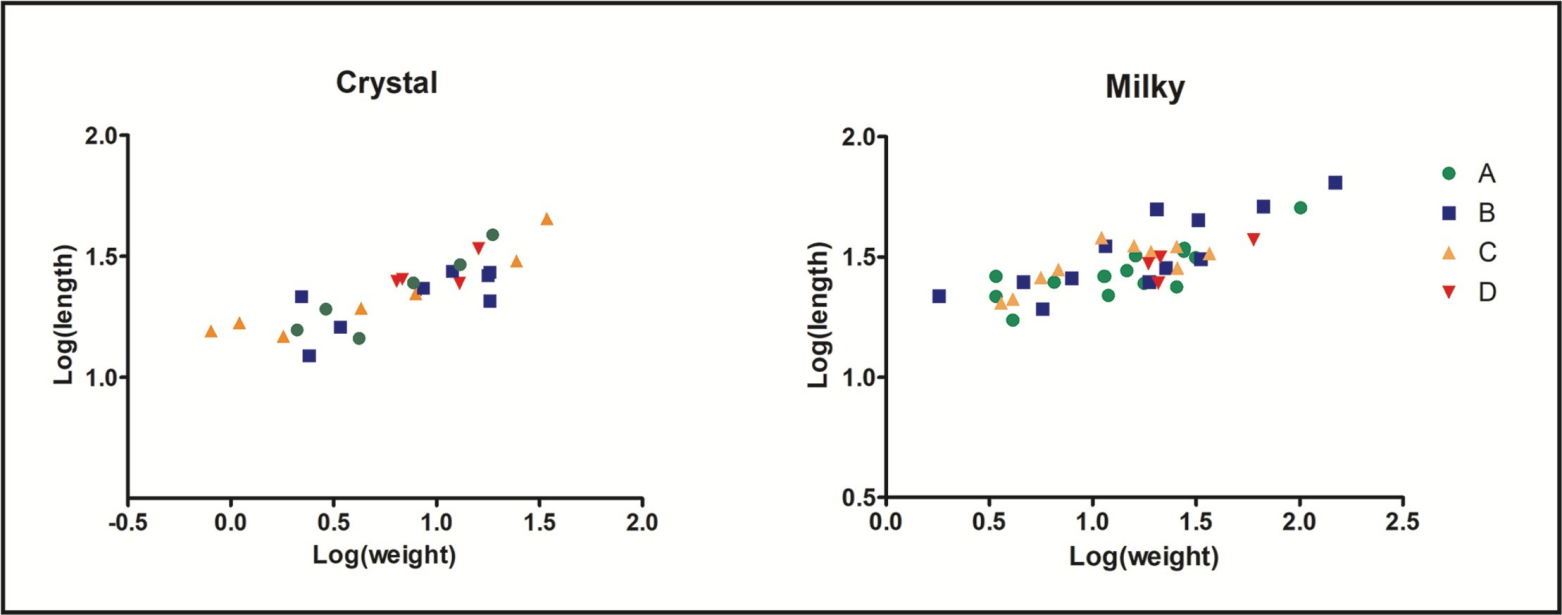
Log transformation plot of weight and length of cores in crystal and milky quartz of Fa-Hien Lena.

Quartz pebbles recovered during the excavation of the site have maximal dimensions of 50-70mm, suggesting that the starting size of raw materials selected in the nearby stream was relatively small. A core with a length of 100mm was recovered in Phase A, implying that larger nodules were occasionally available. Comparison of bipolar core metric attributes suggests that crystal cores were slightly more reduced than artefacts of milky quartz (Table 9, S1 Fig). This characteristic is also evident when assessing the mean length values of complete flakes (S2 and S3 Figs). Blanks in crystal quartz are generally smaller than artefacts in other quartz RMUs throughout the sequence (Tables 5, 7 and 8; S2 and S3 Figs). However, comparing the length and weight values reveals that cores of Phase D are more closely related than the artefacts from other phases, thereby demonstrating different degrees of reduction (Fig 10). This pattern could be explained by the decreasing quality of quartz raw materials after the earliest phases of occupation. Fractures caused by internal flaws and crystalline surfaces could have resulted in knappers discarding or exploiting pebbles in a different manner. The comparison of the length of cortical and non-cortical flakes also shows slightly larger mean values for Phase D than for the other Phases (Tables 5, 7 and 8).

## Discussion

Our analysis of the Fa-Hien Lena lithics has highlighted two major properties of the assemblage: 1) high levels of technological continuity from ~48–45 ka into the Holocene, with lithic technologies focused on bipolar reduction of quartz to produce small flake blanks, and 2) the earliest appearance of backed geometric microliths in South Asia ~48–45 ka. While the number of finished backed microliths identified is low (n = 3) at Fa-Hien Lena, our analysis demonstrates that production is dedicated towards small flake blank manufacture below the regionally appropriate size threshold of 40mm, and thus represent microlithic technologies. Our data highlight that while backing retouch strategies were used at Fa-Hien Lena, the tools themselves may have been transported away from the site for use and discarded elsewhere. This would be in fitting with proposals that microlith tools are associated with projectile technologies during the hunting of arboreal and semi-arboreal game. However, it is also possible that backing may not always have been necessary for successful production of hafted projectiles. Although it has been suggested that the size and type of the raw material could have influenced the adoption of a particular technology [89], the recurrent application of the bipolar concept in the production of the Fa-Hien Lena lithic assemblage could be interpreted as a successful solution for coping with the daily needs for sharp tools rather than an adaptation to the quality of raw material. In Africa, *H*. *sapiens* practiced successful reduction of quartz nodules of small dimensions with different knapping methods from single platform to centripetal and Levallois reduction strategies [71,90–92].

The Late Pleistocene/Holocene foragers of Sri Lanka evidently used other knapping methods (e.g. freehand unidirectional reduction). The bipolar strategy could have been advantageous due to its technological flexibility, allowing a more thorough exploitation of the nodule and the production of relatively large blanks from small cores. Given the primary sourcing of raw materials from small pebbles found in local streams, such efficient reduction would have afforded a suitable supply of flakes. Furthermore, bipolar flakes/blades are generally straight and lacking pronounced bulbs, making them particularly suitable to be hafted onto wooden implements after minimal reshaping of the cutting edges [78]. Resins appropriate for hafting, such as the resin from the *Canarium* sp. trees, have been noted in ethnohistorical record of the Wanniyalaeto, the hunter-gatherer group that is now restricted to the sub-tropical forests of Sri Lanka, but which once was more widespread [1]. Below, we place these findings within their broader regional context.

### Fa-Hien Lena lithic technology in regional context

#### Sri Lanka

Batadomba-lena provides an appropriate local comparison for the lithic technology at Fa-Hien Lena. The site, a small NE facing rockshelter, in gneiss bedrock, is located in Wet Zone lowland rainforest similar to Fa-Hien Lena [1,25,46]. It has also yielded a rich lithic assemblage from occupations commencing *c*. 38,000–36,000 cal. years BP. While we concentrate our discussion on qualitative comparisons of the lithic industries, it is worth noting that both sites have yielded fossil remains of *H*. *sapiens* that are significantly older than those from sites in India, and document specialised rainforest subsistence strategies in the form of rich organic toolkits and faunal assemblages, which are also absent until much later in other regions of South Asia. Unlike Fa-Hien Lena, the Batadomba-lena sequence records a succession of occupations between the Late Pleistocene (Phase 7c, 36–28 ka) (i.e. after Fa-Hien Lena Phase D) to the start of the Holocene (Phase 4a, 12 ka) (contemporary with Fa-Hien Lena Phase C), with no major stratigraphic hiatus [25,70].

At both Fa-Hien Lena and Batadomba-lena, a diverse range of quartz material was exploited as the main raw material for producing small blanks. Although the use of chert is present at both sites, this material is notably absent from the oldest horizons in each sequence. Nevertheless, there are a number of differences in the stone tool technologies between these sites. At Fa-Hien Lena, lithic technology appears to be nearly exclusively based upon bipolar reduction. Although bipolar cores are the most frequent single core type at Batadomba-lena, the combined alternate types of freehand flake cores are more numerous then bipolar cores. Indeed, Lewis and colleagues [33] suggest that, based on their small size, bipolar cores represented the final phase of reduction before discard rather than independent reduction trajectories. Evidence for dedicated blade production at Batadomba-lena is preserved in two cores [33], something absent at Fa-Hien Lena. Finally, retouched artefacts are considerably more numerous in all levels of Batadomba-lena than at Fa-Hien Lena, with geometric microliths evident in all levels alongside other forms of retouched blade and flake tools.

More limited comparisons can be made with the Sri Lankan site of Kitulgala Beli-lena, another Wet Zone rockshelter where a small sample of retouched tools has been studied. At Kitulgala Beli-lena, occupation phases appear from Horizon III (31–26 ka) and continue into the Holocene, with the presence of lithic artefacts alongside organic tools and faunal assemblages [70]. Once again, the small artefact size is highlighted, with the presence of 27 geometric microliths noted from Horizon III, which then occur at lower frequency in younger levels [32,70]. Blade blanks appear to be slightly more frequent than flake blanks for retouched artefacts, although systematic study of larger sample sizes are required [32]. Late Palaeolithic industries are also apparently present beyond the Wet Zone rainforests of Sri Lanka. At the sites of Patirajawela and Bundala [1], which illustrate occupation of sand dunes close to the modern southern coastline beyond the Wet Zone, geometric microliths are reported from sediment contexts radiocarbon dated to between 28–22 ka, while thermoluminescence *terminus post quem* dates of 74–64 ka at Patirajawela remain unconfirmed [1,93].

#### India

The focus on bipolar technology in the earliest levels of Fa-Hien Lena, to the exclusion of other reduction strategies, stands in stark contrast to the oldest known Late Palaeolithic assemblages from India. In southern India, the oldest Late Palaeolithic assemblages come from the rockshelter site of Jwalapuram 9. This site is located at the juncture of forested uplands and a broad lowland river valley, with human occupation dating from >34 ka and persisting through the Late Pleistocene into the early Holocene [48,49]. While the earliest assemblages (Phase E) at Jwalapuram 9 indicate the use of microblade technologies, bipolar methods are absent. Although bipolar technologies only occur in low frequencies during later Phase D (~0.2%) and Phase C (~1%), microblade technologies are present throughout and become more prominent in later levels. Similarities between Jwalapuram 9 and Fa-Hien Lena can be found in the number of final retouched backed artefacts—something absent in the lowest levels but accounting for ~3.6% of the Phase D assemblage and ~4.8% of Phase C which includes backed geometric forms that typically focus on blade blanks [48]. It is notable, however, that bipolar technologies are present in late Middle Palaeolithic assemblages in the immediate vicinity at sites Jwalapuram 20, 21 and 23. At these sites bipolar technologies appear alongside blades and sparse microblades, and there is a frequent use of crystal quartz at Jwalapuram 23 [48].

The oldest Late Palaeolithic industry from central India comes from the open-air site of Mehtakheri in the Narmada valley, dating to ~45 ka [50]. Both microblade and flake reduction sequences are recognised in this assemblage, however, as at Fa-Hien Lena the final number of retouched artefacts is small, consisting of just two backed blades [50]. Neither bipolar technologies nor geometric microliths are present, however. Further evidence for early Late Palaeolithic industries in central India comes from the open-air site of Patne, where the youngest of five assemblages (IIE) is associated with a single radiocarbon date of ~25 ka [47]. However, the site requires renewed and comprehensive dating to place the site and associated lithic assembages in their proper context. At Patne, microlithic tools are reported from assemblages IIB onwards, alongside a range of non-microlithic retouched flake and blade tools, including backed pieces, which appear from the lowest levels of the site. A single bipolar core is reported—this is notably large (length = 51.9mm) in the context of the wider flake and blade core populations at the site [32].

In northern India, dedicated microblade technologies appear from ~55–47 ka at the site of Dhaba 3 in the Middle Son Valley, suggesting an early emergence of Late Palaeolithic industries in the region [52]. Here, Late Palaeolithic industries from open air contexts combine blade and flake reduction trajectories, while backing first appears as a retouching strategy from 42 ka and becomes more prominent by 39–26 ka. However, bipolar reduction methods are not reported from these assemblages while geometric microliths are also not clearly identifiable. The evidence from alluvial sediment sites in eastern India paints a similar picture. Microblade reduction is suggested to appear at Kana ~42 ka and Mahadebbra between ~36–25 ka, geometric backed forms are present at low frequency at Mahadebbra, while no bipolar reduction is evident at either site [53, 94]. Geometric microliths occur in low numbers at occupations of sand dune sites at Buddha Pushkar, western India, as part of a number of assemblages dating to before and after the LGM (28–16 ka) [51]. Backing appears to be a regular feature of a diverse retouched tool kit, while core reduction strategies combine blade, microblade and prepared flake core methods. A number of quartz cores from these assemblages may present evidence for bipolar reduction.

#### A diverse toolkit for diverse adaptations

The process of lithic miniaturisation and the production of small tools was a broad, but diverse phenomenon, appearing more prominently across many regions of Africa and Eurasia during the Late Pleistocene [36,40,41]. In South Africa, microliths appeared in several localities from *c*. 71 ka [95,96], whereas in East Africa backed tools only occur after 50 ka in Tanzania [97], on the coast of Kenya [11], and in Ethiopia [35,98]. Some of the earliest microlith examples, part of the Howiesons Poort techno-complex, have been argued to be short-lived phenomena [99,100]. In Europe, microliths have often been associated with the Upper Palaeolithic from *c*. 45 ka [61], alongside symbolic behaviour, art, and more complex hunting strategies often taken as the hallmark of the arrival of our species [101]. In the Levant, bladelet technologies and the use of backing occur from *c*. 40 ka, with geometric microliths appearing at sites such as Ksar Akil from ~27 ka [102]. Further east, in northern Asia, microblade technologies emerge as part of the ‘Initial Upper Palaeolithic’ *c*. 48 ka [103,104], while in Central Asia geometric microliths and backed bladelets appear as ‘Upper Palaeolithic’ industries from 32 ka [105]. In eastern Asia, microblade technologies emerge in China and the Korean Peninsula from ~30 ka [106,107]. In Southeast Asia, the absence of microlithic technologies until the Holocene is particularly notable [108], and broadly paralleled by the archaeological record of Australia [39] though microliths are known from Pleistocene sites in Queensland and New South Wales [109–111]. Therefore, the archaeological record of Fa-Hien Lena, where lithic technologies targeting small blank sizes and including backed geometric tools appear sometime between 48,000–45,000 cal. years BP, constitutes some of the earliest evidence for microlithic technology outside Africa.

The global emergence of microlilthic technologies appears to be the result of technological convergence, rather than the result of dispersal from a single origin [39]. The diversity of ecological contexts in which microlithic technologies appear to have been independently innovated is particularly startling. In this context, Fa-Hien Lena documents the earliest use of microlithic technologies in tropical rainforest habitats. Long-term lithic technological stability from the Late Pleistocene to the Holocene in tropical contexts is also potentially seen in Southeast Asia. In the territories between Thailand, south China, Vietnam and northwest of Sumatra, the Hoabinhian techno-complex persisted from ~43 to 4 ka [107–110,112–115]. This industry is characterised by plain pebbles, with partially ground edges, choppers, chopping tools, unmodified flakes and the Sumatralith, an oval cobble unilaterally retouched, and short-axes made of transversally fractured tabular cobbles that are also unilaterally retouched [116]. Although the evidence is scarce, probably due to preservation bias, bone points were also documented in the Hoabinhian toolkit after 22 ka [22]. Similar continuity of technological strategies is also observed across island Southeast Asia, at the Niah Caves in Borneo (45–2.5 ka) [2], Jerimalai in East Timor (42–9 ka) [117], and in the Philippine Archipelago from the mid-Late Pleistocene [118].

Appearing in different forms [119], in different environments [33,39,49,70], and sometimes even out of earlier ‘Middle Palaeolithic’ industries [49], microliths are evidently not a marker of rapid expansion of *H*. *sapiens* beyond Africa from *c*. 60 ka. However, their proliferation across Eurasia between *c*. 48–45 ka, in a number of diverse environments, does perhaps still manifest something uniquely human. It was recently proposed that our species was ecologically unique relative to previous members of the genus *Homo*–simultaneously generalising in a diversity of environments while also specialising at the population level in the use of specific resources and landscapes [23]. The diverse temporal appearance of different microlithic forms in sub-Saharan Africa, East Africa, temperate Europe, the Mediterranean, northern and eastern Asia and, now, tropical rainforests is perhaps a material correlate of such a capacity, highlighting the adaptive plasticity of *H*. *sapiens* as it colonised nearly all of the world’s environments during the Late Pleistocene [23,120]. Alongside symbolic material culture [121] and evidence for increased social interaction [17,122], the technological flexibility afforded by microliths may have contributed to a contingent ability to make use of diverse animal and plant resources [123]. While they undoubtedly conferred unprecedented advantages in certain settings, microliths were just one part of what enabled our species to expand and sustain itself in the various ecosystems that have made up its range since the Late Pleistocene.

## Conclusions

Here, we present the first detailed analysis of the earliest microlith assemblage in South Asia (48,000–45,000 cal. years BP), located in tropical rainforest on the island of Sri Lanka. Between *c*. 48–45,000 to 4,000 cal. years BP, despite a long stratigraphic hiatus, technological processes of production and raw material choices show clear continuity, implying a long-term stable adaptation in this part of the world. As old as those found in Europe (~45 ka [61]), the microlith assemblages of Sri Lanka encourage a context-specific approach to these tool types, one that is not limited to savanna, woodland, or coastal plain settings. We argue instead that the recurrent and variable development of microlith technologies is a proxy for the inherent ecological and cultural plasticity of *H*. *sapiens* as it inhabited a diversity of environments and continents on its expansion within and beyond Africa in the Late Pleistocene. It appears likely that, in the Sri Lankan context, microliths may have formed part of composite projectile technologies that enabled the specialised capture of semi-arboreal and arboreal prey; however, more use-wear work is required to confirm this. Technological stability seems to be a feature of tropical rainforest environments in Asia during the Late Pleistocene, potentially highlighting commonalities in the use of lithics in such settings, as well as the possible reliance on tools made from perishable organic materials. Significantly, microliths were clearly a key part of the flexible human ‘toolkit’ that enabled our species to respond–and mediate–dynamic cultural, demographic, and environmental situations [see also 64] as it expanded over nearly all of the Earth’s continents during the Late Pleistocene, in a range currently not evident among other hominin populations.

## Supporting information

S1 Table Total number of lithic artefacts by chronological phase at Fa-Hien Lena Cave. (DOCX)

S1 Fig Comparison of core length and raw material for the different phases of occupation at Fa-Hien Lena Cave. (TIFF)

S2 Fig Comparison of complete cortical flake length and raw material for the different phases of occupation at Fa-Hien Lena Cave. (TIFF)

S3 Fig Comparison of complete flake length and raw material for the different phases of occupation at Fa-Hien Lena Cave. (TIFF)
