# Supplementary material for: Microliths in the South Asian rainforest ~45-4 ka: New insights from Fa-Hien Lena Cave, Sri Lanka
Source: PLoS One. 2019 Oct 2;14(10):e0222606. doi: 10.1371/journal.pone.0222606 (PMC6774521; doi:10.1371/journal.pone.0222606)
Supplement: S1 Table — (DOCX) [file pone.0222606.s001.docx]

| **Phase** | **Layer** | **Flake** | **Bladelet** | **Fragment** | **Debris** | **Tool** | **Core** | **Core Frag.** | **Hammer** | **Total** |
| --- | --- | --- | --- | --- | --- | --- | --- | --- | --- | --- |
| **A** | 1/130 | 18 |  | 35 | 76 |  | 1 |  |  | 130 |
|  | 17/18/19/20/177 | 25 |  | 61 | 31 |  |  | 1 |  | 118 |
|  | 199 | 10 |  | 40 | 42 |  |  |  |  | 92 |
|  | 2/8/9/133/191 | 45 |  | 41 | 83 |  | 1 | 1 |  | 171 |
|  | 39/40/200 | 8 |  | 13 | 8 |  | 1 |  |  | 30 |
|  | 148 | 3 |  | 9 | 8 |  |  |  |  | 20 |
|  | 215 | 9 |  | 21 | 18 |  |  |  |  | 48 |
|  | 216 | 7 |  | 14 | 20 |  | 1 | 2 |  | 44 |
|  | 33 |  |  | 1 |  |  |  |  |  | 1 |
|  | 37 |  |  | 2 | 2 |  |  |  |  | 4 |
|  | 131 | 72 | 3 | 200 | 300 |  | 7 | 6 |  | 588 |
|  | 134 | 110 |  | 241 | 450 |  | 8 | 4 |  | 813 |
|  | 204 |  |  | 2 | 3 |  |  |  |  | 5 |
|  | 3/201 | 14 |  | 27 | 28 |  |  | 1 |  | 70 |
|  | 5/198 | 8 |  | 8 | 4 |  |  |  |  | 20 |
|  | 26 | 2 |  | 11 | 43 |  |  |  |  | 56 |
|  | 31/32/135 | 20 |  | 61 | 28 |  | 3 | 1 |  | 113 |
| **B** | 6/150 | 3 |  | 5 | 2 |  |  |  |  | 10 |
|  | 7/151 | 10 |  | 25 | 15 |  |  | 2 |  | 52 |
|  | 222 | 5 |  | 6 | 1 |  | 1 |  |  | 13 |
|  | 51/152 | 3 |  | 12 | 1 |  | 1 |  |  | 17 |
|  | 52/153 | 5 |  | 19 | 44 |  | 1 | 1 |  | 70 |
|  | 154 | 1 |  | 3 | 1 |  |  |  |  | 5 |
|  | 170 | 7 |  | 7 | 5 |  | 2 | 2 |  | 23 |
|  | 10/65/69/240 | 6 |  | 17 | 7 |  |  |  |  | 30 |
|  | 213 | 13 |  | 16 | 21 |  | 2 |  |  | 52 |
|  | 100 | 1 |  | 7 | 10 |  |  |  |  | 18 |
|  | 102 | 3 |  | 11 | 29 |  |  |  |  | 43 |
|  | 103 | 24 |  | 51 | 86 |  | 1 |  |  | 162 |
|  | 128/116 | 72 | 3 | 287 | 254 |  | 8 | 4 |  | 628 |
|  | 223 | 1 |  | 5 | 13 |  |  |  |  | 19 |
|  | 34/48 | 4 | 2 | 7 | 10 |  |  |  |  | 23 |
|  | 136 | 44 | 1 | 73 | 69 |  |  | 1 |  | 188 |
|  | 38/206 | 15 |  | 31 | 31 |  |  |  |  | 77 |
|  | 205 | 6 |  | 9 | 12 |  |  |  |  | 27 |
|  | 11/217 | 42 |  | 85 | 251 |  | 1 |  |  | 379 |
|  | 22 | 1 |  | 1 | 10 |  |  |  |  | 12 |
|  | 60 | 2 |  |  |  |  |  |  |  | 2 |
|  | 57 |  |  |  |  |  |  |  |  | 0 |
|  | 12/42/72 | 6 |  | 19 | 38 |  |  |  |  | 63 |
|  | 62 |  |  | 4 | 25 |  |  |  |  | 29 |
|  | 137 | 2 |  | 3 | 7 |  |  |  |  | 12 |
|  | 212 |  |  | 1 |  |  |  |  |  | 1 |
|  | 138 | 8 |  | 27 | 54 |  | 3 |  |  | 92 |
|  | 96 | 1 |  | 1 | 2 |  |  |  |  | 4 |
|  | 97 |  |  | 5 | 4 |  |  |  |  | 9 |
|  | 98 | 1 |  | 3 | 1 |  |  |  |  | 5 |
|  | 106 |  |  | 2 | 1 |  |  |  |  | 3 |
|  | 105 | 7 |  | 21 | 40 |  |  |  |  | 68 |
|  | 107 |  |  |  | 7 |  |  |  |  | 7 |
|  | 15 | 1 |  | 3 | 1 |  |  |  |  | 5 |
|  | 13/81 | 19 |  | 33 | 35 |  |  |  |  | 87 |
|  | 99 | 1 |  | 8 | 17 |  |  |  |  | 26 |
|  | 214 | 12 |  | 39 | 158 |  |  |  |  | 209 |
| **C** | 229 | 1 |  | 8 | 27 |  |  |  |  | 36 |
|  | 226 | 1 |  | 2 | 15 |  |  |  |  | 18 |
|  | 225 |  |  | 7 | 18 |  |  |  |  | 25 |
|  | 168 | 9 |  | 23 | 67 |  |  |  |  | 99 |
|  | 237 | 10 |  | 47 | 84 |  | 3 | 1 |  | 145 |
|  | 70/156 | 3 |  | 4 | 16 |  |  |  |  | 23 |
|  | 139/140 | 29 | 1 | 140 | 362 |  | 4 | 4 | 1 | 541 |
|  | 197 |  |  | 1 | 25 |  |  |  |  | 26 |
|  | 141 | 12 |  | 81 | 207 |  | 2 |  | 1 | 303 |
|  | 173 | 7 |  | 26 | 46 |  |  |  |  | 79 |
|  | 242/243 | 6 |  | 23 | 26 |  |  |  |  | 55 |
|  | 232 | 3 |  | 1 | 5 |  |  | 1 |  | 10 |
|  | 142 | 11 |  | 14 | 92 |  | 1 |  |  | 118 |
|  | 143 | 1 |  | 12 | 13 |  |  |  |  | 26 |
|  | 218 | 4 |  | 19 | 26 |  |  |  | 1 | 50 |
|  | 248 | 9 | 1 | 13 | 5 |  |  |  | 1 | 29 |
|  | 174/247 | 74 |  | 212 | 354 |  | 7 | 5 |  | 652 |
|  | 249 | 20 |  | 36 | 40 |  | 1 | 1 |  | 98 |
|  | 250/251 | 15 |  | 50 | 75 |  |  |  |  | 140 |
|  | 144/161 +164 | 23 | 1 | 45 | 104 |  |  | 1 |  | 174 |
|  | 163/235 | 5 |  | 9 | 32 |  |  |  |  | 46 |
| **D** | 14 | 2 |  | 26 | 13 |  |  |  |  | 41 |
|  | 87/115 |  |  | 6 | 128 |  |  |  |  | 134 |
|  | 122 | 11 |  | 7 | 161 |  |  |  |  | 179 |
|  | 88/104/112 | 13 |  | 24 | 41 |  |  |  |  | 78 |
|  | 117 |  |  | 1 |  |  |  |  |  | 1 |
|  | 126 | 6 |  | 17 |  |  |  |  |  | 23 |
|  | 16/16A/89 | 9 | 1 | 14 | 21 |  | 1 |  |  | 46 |
|  | 108 | 10 |  | 31 | 38 |  |  | 1 |  | 80 |
|  | 109 |  |  | 1 | 1 |  |  |  |  | 2 |
|  | 110 | 3 |  | 8 |  |  | 2 |  |  | 13 |
|  | 118 | 12 | 2 | 33 | 5 |  |  |  |  | 52 |
|  | 90/111 | 2 | 1 | 9 | 53 |  |  |  |  | 65 |
|  | 145 | 33 | 3 | 83 | 99 |  | 3 |  |  | 221 |
|  | 166 | 13 |  | 69 | 3 |  |  |  |  | 85 |
|  | 91/119 | 1 |  | 9 |  |  |  |  |  | 10 |
|  | 179 | 20 |  | 16 | 42 |  |  |  |  | 78 |
|  | 175 | 17 |  | 49 | 77 | 1 |  | 2 | 4 | 150 |
|  | 157 | 11 |  | 10 | 17 |  |  |  |  | 38 |
|  | 158 | 28 | 1 | 33 | 2 | 1 | 1 | 3 |  | 69 |
|  | 159 | 28 | 3 | 86 | 3 |  |  | 3 |  | 123 |
|  | 165 | 20 | 1 | 26 | 1 | 1 |  |  |  | 49 |
|  | 146 | 26 |  | 36 | 6 |  |  |  |  | 68 |
|  | 253 | 25 | 1 | 65 | 32 |  | 2 |  |  | 125 |
|  | **Total** | **1210** | **25** | **3065** | **4788** | **3** | **69** | **48** | **8** | **9216** |

S1 Table: Total number of lithic artefacts by chronological phases in Fa-Hien Lena Cave.
