# Supplementary figures and images for: Microliths in the South Asian rainforest ~45-4 ka: New insights from Fa-Hien Lena Cave, Sri Lanka
Source: PLoS One. 2019 Oct 2;14(10):e0222606. doi: 10.1371/journal.pone.0222606 (PMC6774521; doi:10.1371/journal.pone.0222606)

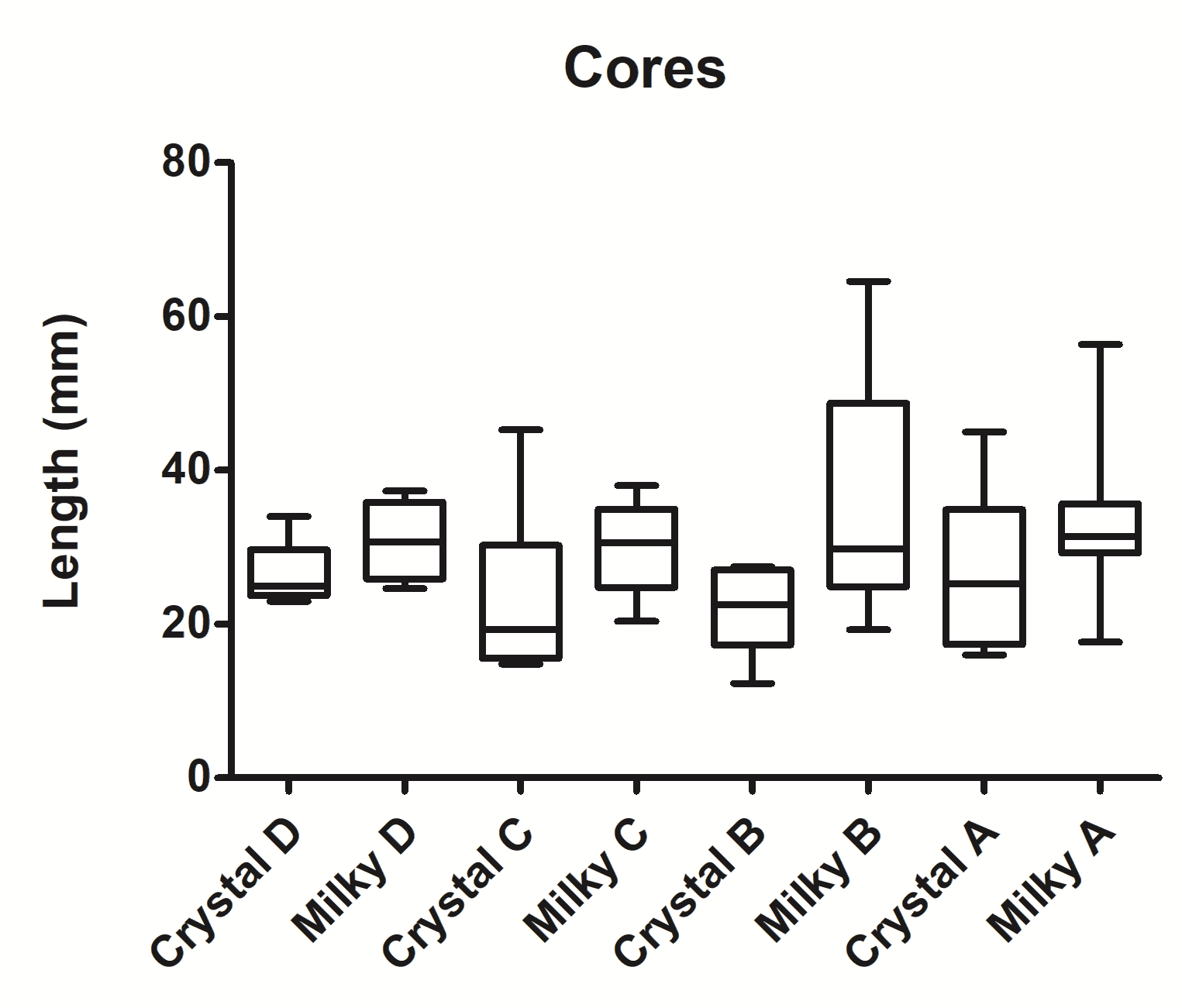

Supplement: S1 Fig — (TIFF) [file pone.0222606.s002.tiff]

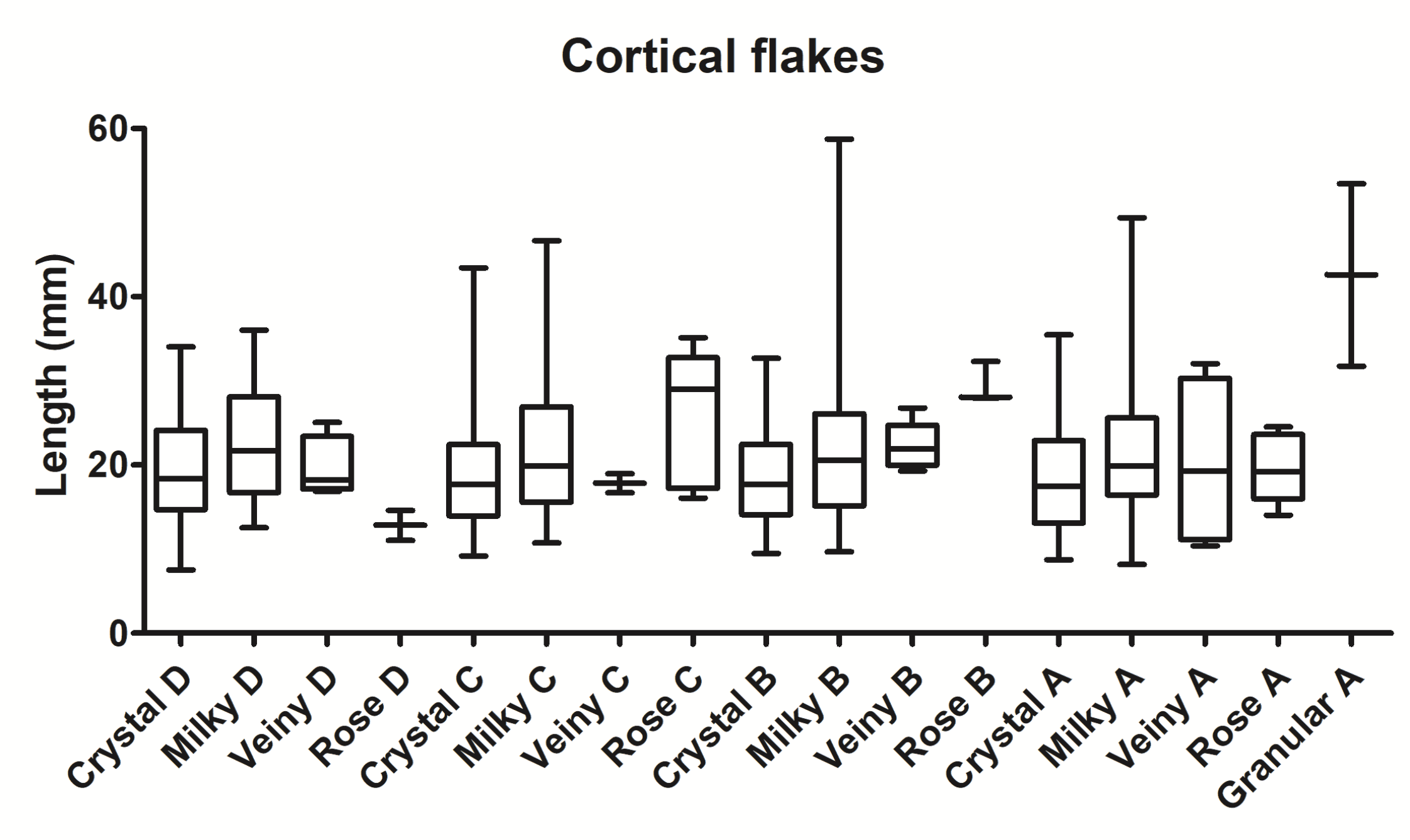

Supplement: S2 Fig — (TIFF) [file pone.0222606.s003.tiff]

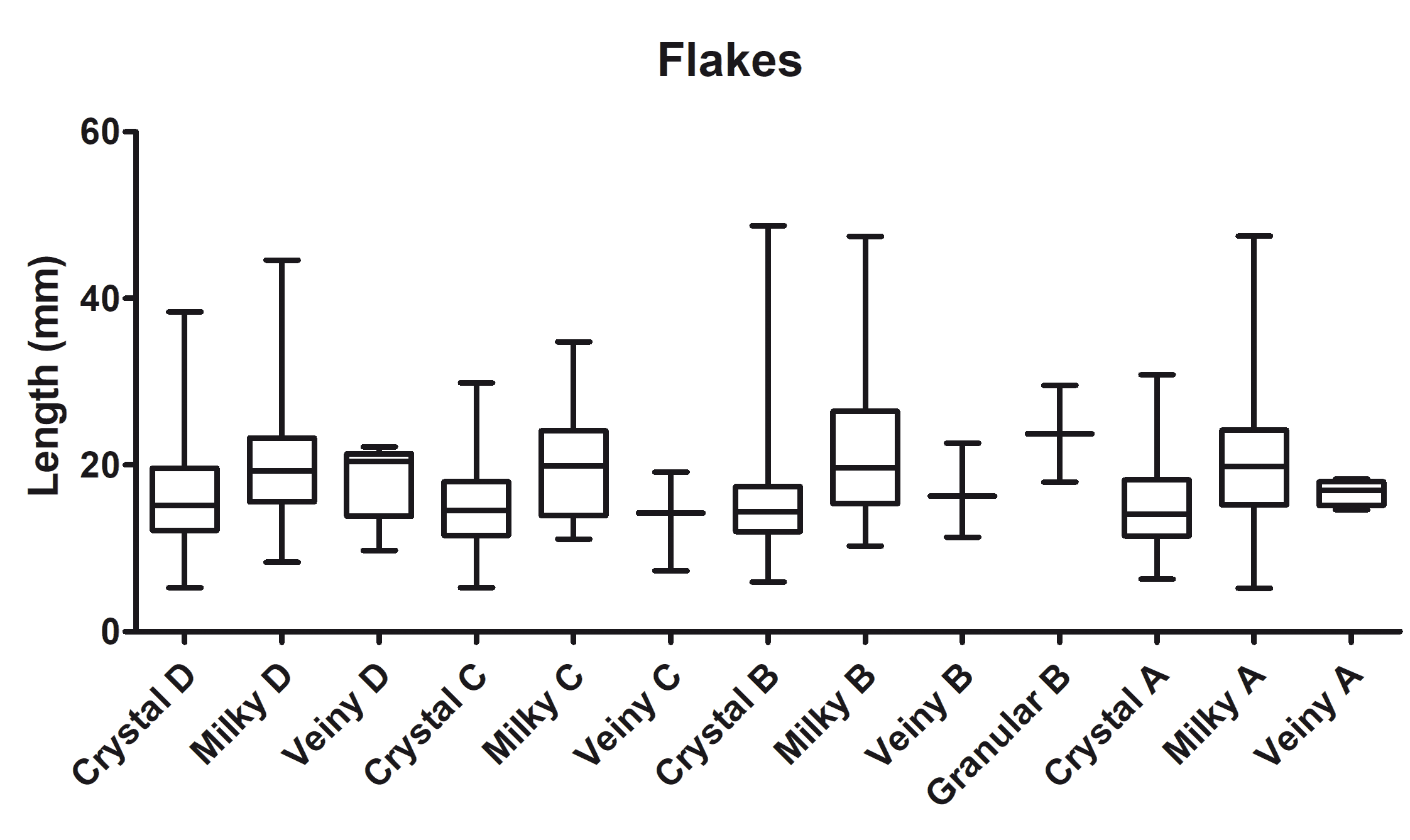

Supplement: S3 Fig — (TIFF) [file pone.0222606.s004.tiff]
